# Synchronization ability of coupled cell-cycle oscillators in changing environments

**DOI:** 10.1186/1752-0509-6-S1-S13

**Published:** 2012-07-16

**Authors:** Wei Zhang, Xiufen Zou

**Affiliations:** 1 School of Mathematics and Statistics, Wuhan University, Wuhan 430072, China

## Abstract

**Background:**

The biochemical oscillator that controls periodic events during the Xenopus embryonic cell cycle is centered on the activity of CDKs, and the cell cycle is driven by a protein circuit that is centered on the cyclin-dependent protein kinase CDK1 and the anaphase-promoting complex (APC). Many studies have been conducted to confirm that the interactions in the cell cycle can produce oscillations and predict behaviors such as synchronization, but much less is known about how the various elaborations and collective behavior of the basic oscillators can affect the robustness of the system. Therefore, in this study, we investigate and model a multi-cell system of the Xenopus embryonic cell cycle oscillators that are coupled through a common complex protein, and then analyze their synchronization ability under four different external stimuli, including a constant input signal, a square-wave periodic signal, a sinusoidal signal and a noise signal.

**Results:**

Through bifurcation analysis and numerical simulations, we obtain synchronization intervals of the sensitive parameters in the individual oscillator and the coupling parameters in the coupled oscillators. Then, we analyze the effects of these parameters on the synchronization period and amplitude, and find interesting phenomena, e.g., there are two synchronization intervals with activation coefficient in the Hill function of the activated CDK1 that activates the Plk1, and different synchronization intervals have distinct influences on the synchronization period and amplitude. To quantify the speediness and robustness of the synchronization, we use two quantities, the synchronization time and the robustness index, to evaluate the synchronization ability. More interestingly, we find that the coupled system has an optimal signal strength that maximizes the synchronization index under different external stimuli. Simulation results also show that the ability and robustness of the synchronization for the square-wave periodic signal of cyclin synthesis is strongest in comparison to the other three different signals.

**Conclusions:**

These results suggest that the reaction process in which the activated cyclin-CDK1 activates the Plk1 has a very important influence on the synchronization ability of the coupled system, and the square-wave periodic signal of cyclin synthesis is more conducive to the synchronization and robustness of the coupled cell-cycle oscillators. Our study provides insight into the internal mechanisms of the cell cycle system and helps to generate hypotheses for further research.

## Background

Oscillations play a vital role in many dynamic cellular processes, and two typical examples of genetic oscillators are the cell cycle oscillators [1,2] and circadian clocks [3]. Understanding the molecular mechanisms that are responsible for oscillations and their collective behaviors is important for clarifying the dynamics of cellular life and for designing efficient drug doses. Synchronization is a type of typical collective behavior and is a basic motion in nature that can explain many natural phenomena [4,5]. Recent studies have shown that cellular communication is accomplished by synchronization, and a number of simulations and fundamental experimental studies have also confirmed synchronization mechanisms in some interacting or independent biological systems [6-9]. The revealed synchronization mechanisms and the dynamics of control in multi-cellular systems are essential for understanding inherent mechanisms of living organisms at both the molecular and cellular levels [10-12].

The biochemical oscillator that controls periodic events during the Xenopus embryonic cell cycle is centered on the activity of CDKs, and the cell cycle is driven by a protein circuit that is centered on the cyclin-dependent protein kinase CDK1 and the anaphase-promoting complex (APC). Many studies have been conducted that confirm that the interactions in the cell cycle can produce oscillations and predict behaviors such as synchronization [13-16], but much less is known about how the various elaborations and collective behavior of the basic oscillators can affect the robustness of the system and how cells use the information to control the cell cycle.

The experiments indicated that the cyclin-dependent kinases (CDKs) are not solely responsible for establishing the global cell-cycle transcription program, although they have a function in the regulation of cell cycle transcription, and the precise cell cycle could be controlled by coupling a transcription factor network oscillator with the cyclin-CDK oscillator [13]. To elucidate various synchronization mechanisms (from the viewpoint of the dynamics) by investigating the effects of various biologically plausible couplings and external stimuli, in this paper we use the three-order ordinary differential equation (ODE) model of the Xenopus embryonic cell cycle that was presented in the literature [1] as a basic model for one oscillator and study the synchronization for a network of N oscillators in which all of the units were indirectly coupled by interacting with a common environment. We present the coupled model of cell cycle oscillators and the synchronization feature of the coupled system, and we determine the synchronization intervals of system parameters and analyze the effects of parameters on the period and amplitude when synchronization is achieved.

Furthermore, the recent biological experiments found that cell cycle oscillations in Xenopus early embryonic extracts might not be driven by constant cyclin B synthesis ([17] and [18]). Therefore, we consider the cyclin synthesis rate as four possible impulse signals, including a constant input signal, square-wave periodic signal, a sinusoidal signal and a noise signal, and investigate the synchronization ability under different external stimuli by defining two measures, including the synchronization time and the robustness index. These studies are viewed as an important step toward the comprehensive understanding of mechanisms of the Xenopus embryonic cell cycle.

## Results

### Synchronization of a population of N-cell cycle oscillators

For simplicity, we analyze the case of ten identical oscillators (N = 10), and the same results can be obtained when N is set to be greater than 10. By the numerical simulation, all of the parameters of the coupled system that can reach synchronization are obtained (Table 1), and the synchronization diagram (Additional file 1). The oscillation period of the coupled system is approximately 4.315 min when synchronization is achieved, and the period of a single oscillator is approximately 3.78 min.

**Table 1:** The parameter settings of the coupled system

*α_1_*	*α_2_*	*α_3_*	*β_1_*	*β_2_*	*β_3_*	*K_1_*	*K*_2_	*K_3_*
0.1	3	3	3	1	1	0.5	0.5	0.5

***n_1_***	***n_2_***	***n_3_***	***n***	***k_0_***	***K_a_***	***k_m_***	***k***	***K_L_***

4	4	4	3	2	0.5	1.5	1	0.5

### Parameter sensitivity analysis of the coupled system

The range of the parameter distributions is set to be a random number between [0, 1], and we obtain an average over 100 runs. All of the results are normalized, and the effects of the parameter changes on the amounts of the three variables and the complex protein R in equation (2) (Additional file 2). From Additional file 2 we can see that the most sensitive parameter is K_1_, followed by α_1_, K_a_, K_2_, K_3_, β_2_, β_3_, α_3 _and k_m_.

### Synchronization intervals for the selected parameters

The bifurcation diagram for the parameters of the variations in the complex protein CDK1 (C_1_) of the first oscillator in the coupled system (Additional files 3, 4, 5). From Additional file 3, we find an interesting phenomenon, which is that there are two stable states for parameters K_2 _(Additional file 3 (B)) and β_2 _(Additional file 4 (A)), when K_2 _varies in [0, 0.8] and when β_2 _varies in [0, 2], respectively.

Furthermore, we searched for the synchronization intervals of these parameters through numerical simulations. We assume that the system achieves synchronization when the synchronization error is smaller than 1e-5. The synchronization intervals obtained for the parameters are shown in Table 2.

**Table 2:** The synchronization intervals for the sensitive parameters

*K_1_*	*K_2_*	*K_3_*	*α_1_*	*α_3_*	*β_2_*
[0.48, 0.57]	[0.185, 0.22] [0.48, 0.57]	[0.48, 0.57]	[0.09, 0.21]	[2.2, 3.5]	[0.9, 1.3]
***β_3_***	***k_m_***	***K_L_***	***K_a_***	***k***	***k_0_***

[0.89, 1.3]	[1.3, 1.6]	[0.44, 0.55]	[0.46, 0.52]	[0.92, 1.3]	[1.85, 2.3]

From Table 2 we can see that there are two synchronization intervals for K_2_, and the other parameters have only one synchronization interval. Although there are two stable states for the degradation rate β_2_, there is only one synchronization interval. We can also observe that the more sensitive parameters have smaller synchronization intervals.

**Figure 1:**
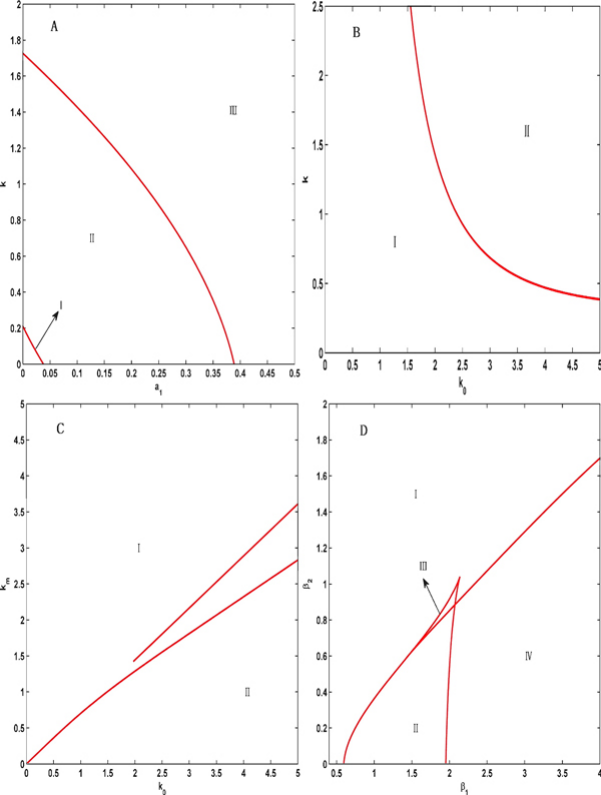
**Two-parameter bifurcation diagrams for four groups of parameters**. (A) The bifurcation diagram for the coupling strength k and the synthetic rate *α*_1_. The whole region is divided into three regions I, II and III. I and III: stable regions. II: oscillation region. (B) The bifurcation diagram for the coupling strength k and the coefficient k_0_. I: oscillation region. II: stable region. (C) The bifurcation diagram for the degradation rate k_m _and the coefficient k_0_. I: oscillation region. II: stable region. The behavior of the system in the region between two lines is unclear. (D) The bifurcation diagrams for the degradation rates *β*_1 _and *β*_2_. I, II and III: stable regions. Region IV: oscillation region.

**Figure 2:**
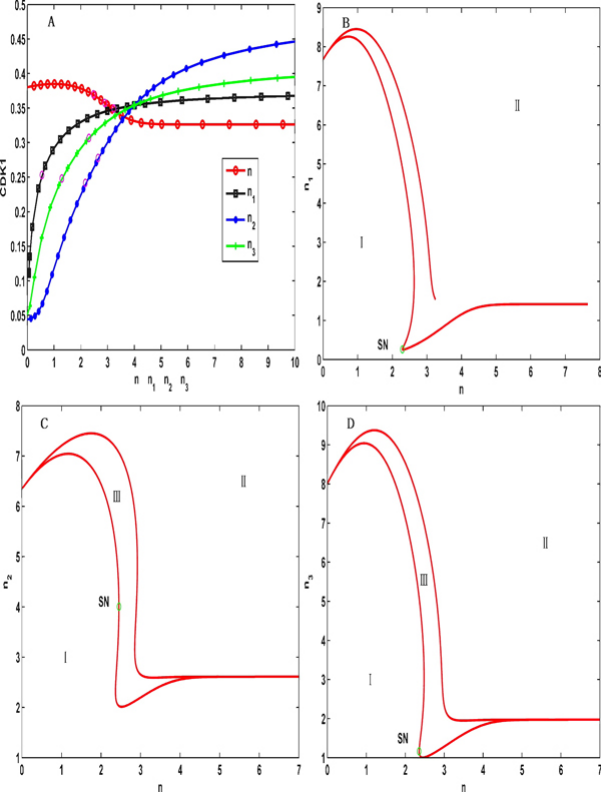
**Bifurcation diagrams for the Hill coefficients**. (A)The single parameter bifurcation diagram of n, n_1_, n_2 _and n_3_, with an increase of the parameter n the concentration of CDK1 decreases slightly but the concentration of CDK1 increases gradually with increases in n_1_, n_2 _and n_3_. The changing of n_2 _is very sensitivity to the concentration of CDK1. (B)(C) and (D) are two-parameter bifurcation diagrams for each pair among n, n_1_, n_2 _and n_3_, respectively. I: stable region, and II and III: oscillation regions.

To further investigate the dynamical features of the system in the synchronization intervals, we provide some two-parameter bifurcation diagrams with the XPPAUT software [19]; these diagrams are shown in Figures 1 and 2. Figure 1 further demonstrates an oscillation feature in the determined synchronization intervals. Figure 2 shows that the oscillation system can reach synchronization as long as the Hill coefficients n_1_, n_2 _and n_3 _are greater than 3 when the coupled Hill coefficient n is set to be no smaller than 3, indicating the rationality of the parameter settings for the Hill coefficients in Table 1.

### The effects of sensitive parameters on the synchronization period and amplitude

#### (A)The effects of the activation coefficients K_1_, K_2_, and K_3 _in the Hill functions

From Additional file 6 we can observe that the activation coefficients K_1 _and K_3 _have the same influence on the period and amplitude, which is that the oscillation period and amplitude are almost linearly decreased with increases in K_1 _and K_3_.

However, the activation coefficient K_2 _has distinct influences on the period and amplitude of the synchronization system in different synchronization intervals (Additional file 7). In the first interval [0.185, 0.22], the period increases and the amplitude is almost the same, but in the second interval, the period and amplitudes decrease.

**Figure 3:**
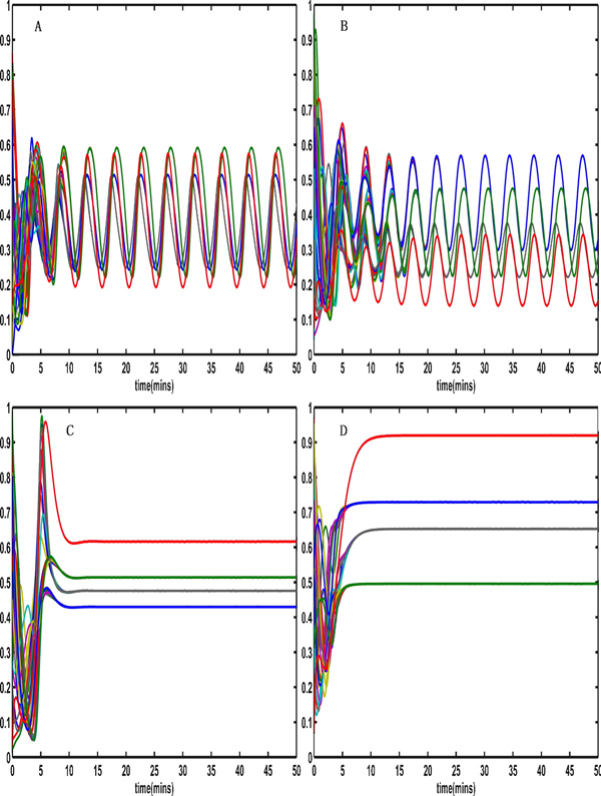
**The coupled system switches from stable period oscillations to the stable steady state**. (A) The coupled system switches from stable period oscillation when α_2 _= 1.6 to the stable steady state (C) when α_2 _= 1, the other parameters are set as Table 1. The coupled system switches from the stable period oscillation (B) when α_3 _= 1.6 to the stable steady state (D) when α_3 _= 1.2.

When K_2 _varies in the interval [0.35, 0.42], and the parameter α_2 _changes from 1.6 to 1.0 (Figure 3(A) and Figure 3(C)) or α_3 _changes from 1.6 to 1.2 (Figure 3(B) and Figure 3(D)), the coupled system switches from stable period oscillations to a stable steady state (Figure 3).

**Figure 4:**
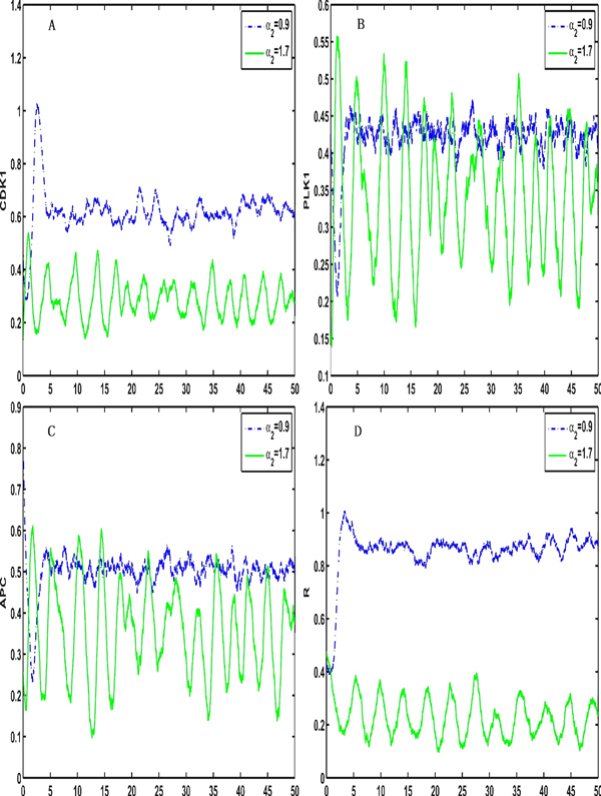
**The coupled system switch from a stable steady state to stable period oscillations when intrinsic noises are added and the parameter α_2 _is changed**. The coupled system switches from a stable steady state to stable period oscillations if inner noises are introduced when K_2 _is set to be between two different synchronization intervals (The intensity of inner noise is 0.001 and the parameter α_2 _changes from 0.9 to 1.7)

**Figure 5:**
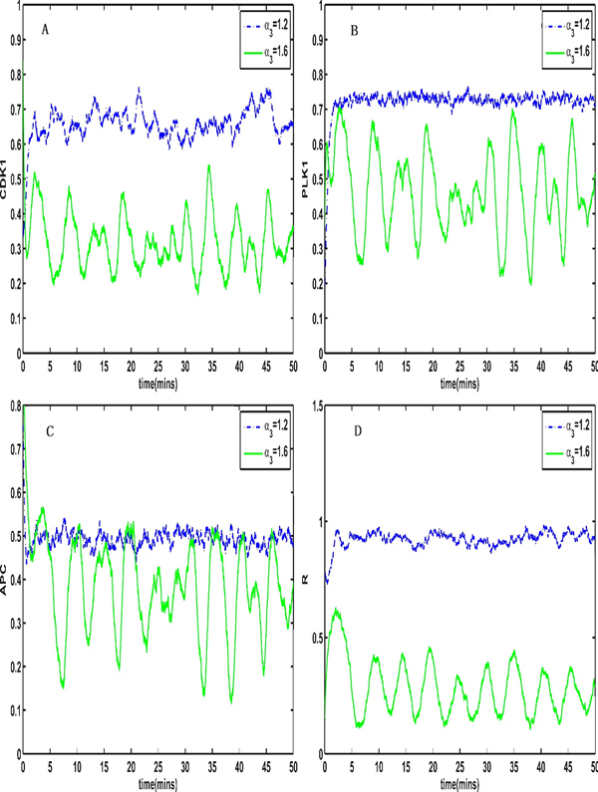
**The coupled system switch from a stable steady state to stable period oscillations when intrinsic noises are added and the parameter α_3 _is changed**. The coupled system switches from a stable steady state to stable period oscillations if inner noises are introduced when K_2 _is between two different synchronization intervals (The intensity of inner noise is 0.001 and the parameter α_3 _changes from 1.2 to 1.6)

To consider the influence of noise on the features of the system, we introduce the inner noise in the system (2). Figure 4 and 5 show stochastic transitions between the stable steady state and the stable limit cycle when the intensity of the inner noise is 0.001 and the parameter α_2 _changes from 0.9 to 1.7 or the parameter α_3 _changes from 1.2 to 1.6, respectively, indicating that the coupled system can switch between a stable state and a stable periodic orbit regardless of whether there is noise.

#### (B)The effects of α_1 _and α_3 _on the period and amplitude when synchronization is achieved

The simulated course of the period and amplitude with changes in α_1 _and α_3 _are depicted (Additional file 8). From Additional file 8 we can see that the oscillation period and amplitude decreased with an increase in α_1 _and increase with an increase in α_3_, but the change of the period for both α_1 _and α_3 _is obvious and the change in the amplitude is slight. This observation further demonstrates that the activation rates can adjust the oscillation period in the coupled system, which is the same as in the single oscillator of interlinked positive and negative feedback [20].

#### (C) The effects of coupling parameters on the period and amplitude when synchronization is achieved

The effects of the coupling strength k, the ratio coefficient k_0 _and the activation coefficients K_L _and K_a _on the period and amplitude are shown in Additional files 9 and 10. With an increase in these parameters, the oscillation periods for parameters K_L_, K_a _and K increase, but the oscillation period for parameter K_0 _decreases. The trend of the oscillation amplitudes is similar to the periods except for the coupling strength k. However, the influence of the coupling parameters on the period is greater than the influence on the amplitude, especially for the activation coefficient K_a _of the Hill function of C_i_.

### Comparisons of synchronization abilities based on the synchronization time and robustness index

**Figure 6:**
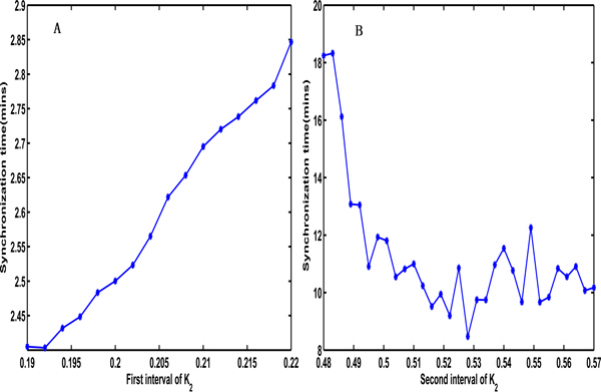
**The effects of parameter K_2 _on the synchronization time in two different synchronization intervals**. (A) The synchronization time of K_2 _in the first synchronization interval. It shows the synchronization time increases with an increase in K_2_. (B) The synchronization time of K_2 _in the second synchronization interval. It shows the synchronization time decreases with an increase of K_2_.

To evaluate the synchronization ability of a coupled system, we simulated two metrics, the synchronization time and the robustness index (see Methods). First, we analyzed the effect of K_2 _on the synchronization time (Figure 6); we found that the synchronization time increased with an increase of K_2 _in the first interval and the synchronization time decreased with an increase of K_2 _in the second interval. We also observed that the synchronization time in the first interval was much shorter than the synchronization time in the second interval, but the synchronization was very sensitive to changes in the initial values.

**Figure 7:**
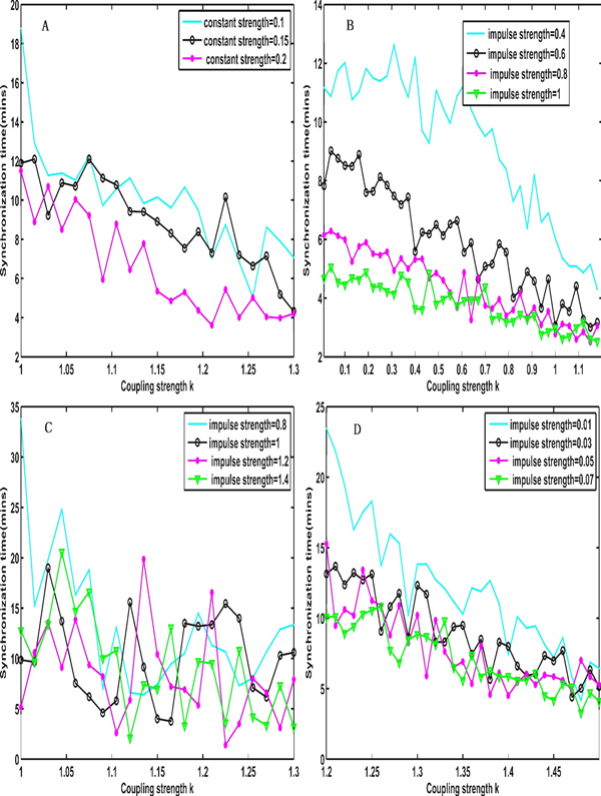
**The effects of the coupling strength on the synchronization time under different impulse signals and impulse strengths**. (A) The effects of the coupling strength k on the synchronization time under a constant signal input with the impulse strengths 0.1, 0.15 and 0.2, respectively. (B) The effects of the coupling strength k on the synchronization time under a square wave signal input with the impulse strengths 0.4, 0.6, 0.8 and 1, respectively. (C) The effects of the coupling strength k on the synchronization time under a sine signal input with the impulse strengths 0.8, 1, 1.2 and 1.4, respectively. (C) The effects of the coupling strength k on the synchronization time under the Gauss noise input with the mean strengths 0.01, 0.03, 0.05 and 0.07, respectively and standard deviation 0.001. Where the constant signal: the cyclic synthesis α_1 _= aq. The square wave signal, the sine signal and the noise signal are corresponding to the following formula: α1t=aqift modt0<t10otherwise, $\alpha_{1}\left(\text{t}\right)=\left\{\begin{array}{cc} \text{a}q\text{sin}\left(t\right) & if\text{sin}\left(t\right)>0\\ 0 & otherwise \end{array}\right)$, $\alpha_{1}\left(\text{t}\right)=\text{aq}+\text{bq}*rand$. Where aq is the signal strength, t_0 _is set to 4 and t_1 _is set to 2 in the square wave signal, bq is set to 0.001 when aq is 0.01 and bq is set to 0.01 when aq is larger than 0.01 in the noise signal.

Figure 7 shows how the different signal strengths affect the synchronization time with an increase in the coupling strength under a constant input signal, a square-wave periodic signal, a sinusoidal signal and a noise signal, respectively. From this figure, we can observe that the synchronization time decreases with a growth in the impulse strength and a coupling strength under a constant signal (Figure 7(A)), a square-wave periodic signal (Figure 7(B)) and a noise signal (Figure 7(D)), which indicate that the synchronization ability is improved when the coupling strength and the impulse strength are increased and is most obvious under a square-wave periodic signal. However, the synchronization time has no clear trend with a change in the coupling strength and the impulse strength if the cyclin synthesis is the sinusoidal signal (Figure 7(C)), which indicates that the coupling strength and impulse strength have no obvious influence.

**Figure 8:**
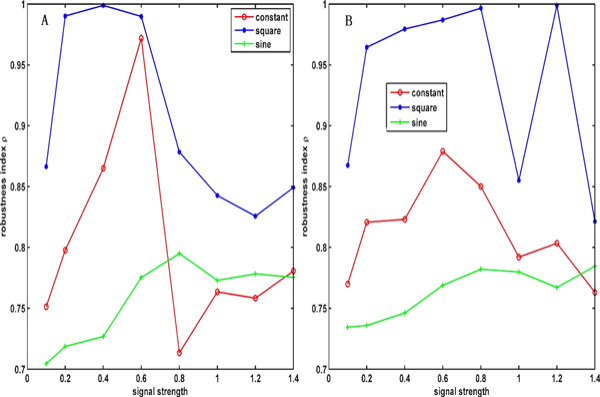
**The effects of the signal strength on the robustness in three different signal inputs under the variation of parameters at 10% and 20%**. (A) The effects of the signal strength on the robustness index under a constant signal, a square wave signal and a sine signal at the variation of 10%. (B) The effects of the signal strength on the robustness index under a constant signal, a square wave signal and a sine signal at the variation of 20%.

**Figure 9:**
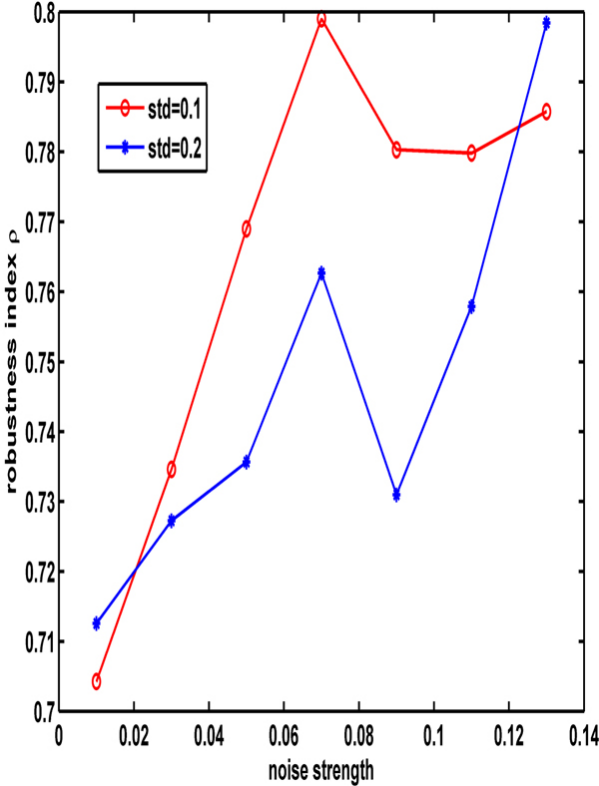
**The effects of the signal strength on the robustness in noise signal input under the variation of parameters at 10% and 20%**. The legend with circle and star lines represent the variation of 10% and 20%, respectively.

Figure 8 displays the robustness indexes under three different signals: a constant input signal, a square-wave periodic signal and a sinusoidal signal with an increase in the signal strength under a variation in the parameters of 10% (Figure 8(A)) and 20% (Figure 8(B)). This figure shows that the robustness of the square-wave periodic signal is the strongest of all of the signals, regardless of how much the variation of the parameters is. Figure 9 depicts the effects of the noise strength for the noise signal on the robustness index under two types of variation strengths of parameters, indicating that the relatively small variation of parameters is benefited to the robustness of the system under the noise signal.

## Discussion

In this study, we investigated the synchronization feature of one coupling system of N cell-cycle oscillators that were coupled through a common complex protein. The work of Mclsaac. R et al. [21] analyzed the spatial synchronization oscillation of Xenopus embryos that was triggered by the fertilization-initiated calcium wave; this investigation may offer insights into determining the components of the complex protein R.

**Figure 10:**
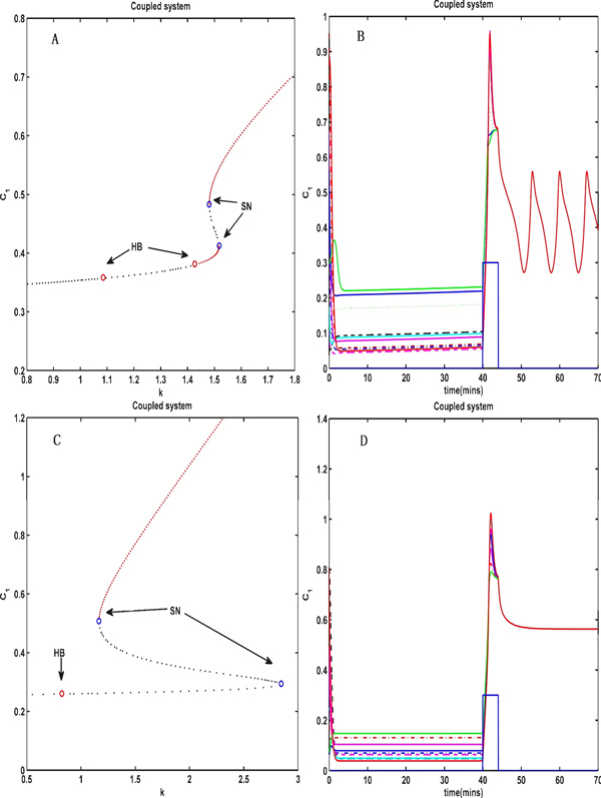
**The bifurcation diagram for the coupling strength k**. (A) Bifurcation diagram for the coupling strength k. When the coupling strength is increased to the range between two saddle-node bifurcations, the coupled system can exhibit bistability and also exhibits some hysteresis, i.e., CDK1 converges to a low or a high state depending on the initial conditions. But when k is set to 1.6 and the synthetic rate *α*_1 _of CDK1 is changed into the square wave signal, the coupled system behaviors as (B): a pulse input drives CDK1 into the upper state and then oscillates, or oscillates all the time which depends on the initial state of the coupled system. (C) The bifurcation diagram for k when K_2 _= 0.35, *α*_3 _= 1.6 and other parameters are set as Table 1. (D) shows that the pulse input drives CDK1 into the upper state and cannot get back. SN: Saddle-Node point. HB: Hopf bifurcation point.

The cell division cycle of the Xenopus embryo was demonstrated to consist of two phases: interphase and metaphase [21], which are characterized by low and high levels of CDK1 activity, respectively. In the work of Pomerening et al. [18], a numerical model of the embryonic cell-cycle network was developed, in which the CDK1 activity was increased by dual positive feedback, while the CDK1 activity was reduced by a single negative feedback to explain the possible underlying bistability of the network. We can also observe a similar phenomenon in the coupled model through the bifurcation analysis of the coupling strength k (Figure 10). From Figure 10, we can see that the coupled system can exhibit bistability when the coupling strength k is increased to the region between two saddle-node bifurcations. We can see that the coupled system exhibits some hysteresis, i.e., CDK1 converges to a low or high state depending on the initial condition, which is a specific feature of the coupled system.

There are also some limitations to our approach. In our proposed coupled model, we chose three components, which composed a negative feedback loop as the basic model; this configuration captured the main features of the cell cycle but may have limitations for interpreting the details of the mechanism of the cell cycle, for example, adding the positive feedback of the Wee1 as well as Cdc25 on the cyclin CDK1 may contribute a more widely tunable period and amplitude of the oscillation [18].

Although we have mainly examined effects of the most sensitive parameters and coupled parameters on the cellular dynamics, there are also other important factors that may play important roles in biological processes and should be further investigated from theoretical viewpoints.

## Conclusions

In this paper, a new dynamical global coupled model for cell cycle oscillators is presented. Through bifurcation analysis and numerical simulations, we determined synchronization intervals of the coupled system. Our simulation results show that the more sensitive parameters have smaller synchronization intervals. Furthermore, we find that there are two synchronization intervals of the activation coefficient in the Hill function of the activated CDK1 that activate the Plk1, and different synchronization intervals have distinct influences on the period and amplitude of the synchronization system. Afterwards, when this parameter shifts from two different synchronization intervals, the coupled system can switch from stable period oscillations to a stable steady state. Computational results through the two metrics, the synchronization time and the robustness index, indicate that a larger coupling strength has a shorter synchronization time for the three signals, and the robustness index for the square-wave periodic signal of cyclin synthesis is strongest in comparison to the other signals. These results suggest that the reaction process in which the activated cyclin-CDK1 activates the Plk1 has a very important influence on the synchronization features of the coupled system. The square-wave periodic signal of cyclin synthesis is more beneficial to the synchronization and robustness of the coupled cell-cycle oscillators.

Our work not only can be viewed as an important step toward the comprehensive understanding of the mechanisms of the Xenopus embryonic cell cycle but also can provide a guide for further biological experiments.

## Models and methods

### Model of coupled cell cycle regulatory oscillators

**Figure 11:**
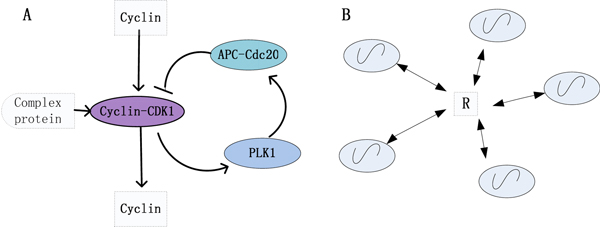
**The simplified diagrams of the Xenopus Embryonic cell cycle and global coupling style between oscillators**. (A) The simplified diagrams of the Xenopus Embryonic cell cycle (redrawn from [1]). (B) Global coupling style between N oscillators.

The simplified reaction diagram of the Embryonic cell cycle is depicted in Figure 11(A). The cyclin-dependent protein kinase CDK1 is activated by the rapid, high-affinity binding of cyclin, and forms the synthesized protein Cyclin-CDK1, which is the master regulator of mitosis. A protein such as Polo-like kinase (Plk1) cooperates with cyclin-CDK1 to activate the E3 ubiquitin ligase APC-Cdc20, and APC-Cdc20 inactivates cyclin-CDK1.

For cell *i*, we assume that CDK1 is activated by a constant rate of cyclin synthesis (α_1_), and the inactivation rate is proportional to the concentration of CDK1* (C_i_) times a Hill function of APC*(A_i_). The activation of Plk1 (P_i_) by CDK1* is proportional to the concentration of inactive Plk1 (we also assume the total concentration of active and inactive Plk1 to be constant, specifically 1-P_i_) times a Hill function of CDK1*(C_i_), and the inactivation is proportional to Plk1*(P_i_). The activation of APC (A_i_) by Plk1 is proportional to the concentration of inactive APC (1- Ai) times a Hill function of Plk1 (Pi), and the rate of inactivation of APC is described by simple mass action kinetics. The resulting three-ODE model is the following:

(1) $$\begin{array}{c} \frac{dC_{\text{i}}}{dt}=\alpha_{1}-\beta_{1}C_{i}\frac{{A_{i}}^{n_{1}}}{{K_{1}}^{n_{1}}+{A_{i}}^{n_{1}}}\\ \frac{dP_{i}}{dt}=\alpha_{2}\left(1-P_{i}\right)\frac{{C_{i}}^{n_{2}}}{{K_{2}}^{n_{2}}+{C_{i}}^{n_{2}}}-\beta_{2}P_{i}\;\;\;\\ \frac{dA_{i}}{dt}=\alpha_{3}\left(1-A_{i}\right)\frac{{P_{i}}^{n_{3}}}{{K_{3}}^{n_{3}}+{P_{i}}^{n_{3}}}-\beta_{3}A_{i} \end{array}$$

where the parameters α_i_, β_i _(i = 1, 2 and 3), K_1_, K_2 _and K_3 _are set to be the same as those in the Literature [1], except for the Hill coefficients n_1_, n_2 _and n_3_, which are set to be 4.

To reveal the internal mechanism of the Xenopus embryonic cell cycle, we assume that all of the cells are coupled indirectly through the common extracellular medium, in other words, they are coupled through a complex protein (R) that excites the protein of Cyclin-CDK1 in the core cell cycle regulatory pathway. The diagram for global coupling of the cell cycle oscillators is shown as in Figure 11(B).

The ODE equations for N cell oscillators (denoted by i = 1, 2,.., N) are written as follows:

(2) $$\begin{array}{c} \frac{dC_{\text{i}}}{dt}=\alpha_{1}-\beta_{1}C_{i}\frac{{A_{i}}^{n_{1}}}{{K_{1}}^{n_{1}}+{A_{i}}^{n_{1}}}+k\frac{R^{n}}{{K_{L}}^{n}+R^{n}}\\ \frac{dP_{i}}{dt}=\alpha_{2}\left(1-P_{i}\right)\frac{{C_{i}}^{n_{2}}}{{K_{2}}^{n_{2}}+{C_{i}}^{n_{2}}}-\beta_{2}P_{i}\;\;\;\;\\ \frac{dA_{i}}{dt}=\alpha_{3}\left(1-A_{i}\right)\frac{{P_{i}}^{n_{3}}}{{K_{3}}^{n_{3}}+{P_{i}}^{n_{3}}}-\beta_{3}A_{i}\;\;\\ \frac{dR}{dt}=\frac{k_{0}}{N}\overset{N}{\underset{i=1}{\sum }}\frac{{C_{i}}^{n}}{{K_{a}}^{n}+{C_{i}}^{n}}-k_{m}R \end{array}$$

### Synchronization of a population of N-cell cycle oscillators

In order to quantify the level of synchronization of the coupled system, we introduce the synchronization error proposed in [22] as follows.

$$E\text{ = }\frac{\text{1}}{\text{N}}\overset{N}{\underset{i=2}{\sum }}\left[{\left(C_{i}-C_{1}\right)}^{2}+{\left(P_{i}-P_{1}\right)}^{2}+{\left(A_{i}-A_{1}\right)}^{2}\right]$$

The coupled system is defined to achieve synchronization when E reaches zero in a limited amount of time. In our simulation, we assume that the system achieves synchronization when the synchronization error E is smaller than 1e-5.

### Parameter sensitivity analysis of the coupled system

To investigate the effects of parameter changes on the amount of all of the variables in the coupled system, we make the sensitivity analysis of parameters with an approach proposed in [23]. For the continuous state equation that has continuous first-order partial derivatives with parameters λ_0_, we have the following:

(4) $$\begin{array}{c} \frac{dx}{dt}=f\left(t,x,\lambda_{0}\right)\\ x\left(t_{0}\right)=x_{0} \end{array}$$

The solution can be approximated by expanding the Taylor series about the nominal solution *x*(*t, λ*_0_).

(5) $$x\left(t,\lambda \right)\approx x\left(t,\lambda_{0}\right)+S\left(t\right)\left(\lambda -\lambda_{0}\right)$$

The sensitivity function *S (t) *provides the first-order estimates of the effects of the parameter variations on the solutions. When all of the values λ are in a small ball centered at λ_0_, the sensitivity function suffices to approximate the solution. Then, we can calculate the sensitivity of the system parameters by solving the following sensitivity equation (See [23] for details):

(6) $$\frac{dS\left(t\right)}{dt}=\left[\frac{\partial f\left(t\;,x,\lambda \right)}{\partial x}\right]{|}_{\lambda =\lambda_{0}}S+\left[\frac{\partial f\left(t\;,x,\lambda \right)}{\partial \lambda }\right]{|}_{\lambda =\lambda_{0}},S\left(t_{0}\right)=0\;\;\;$$

The range of the parameter distributions is set to be a random number between [0, 1] and we obtain an average over 100 runs; all of the results are normalized.

### Identification of the synchronization intervals for the selected parameters

To analyze the effects on the synchronization when the parameters change, we perform a bifurcation analysis for the sensitive parameters and the coupling parameters by varying the chosen parameter and fixing the other parameters.

### Calculation of the synchronization time and robustness index

To quantify the speediness and robustness of the synchronization, we use two quantities, the synchronization time and robustness index, to evaluate the synchronization ability under different conditions. The synchronization time is calculated according to the time when the synchronization error of the coupled system is smaller than 1e-5. The robustness index (r) is computed with the following formula, which is similar to the formulas in [24,25].

(7) $$r=\frac{1}{N{\text{log}}_{2}N}\overset{M}{\underset{k=1}{\sum }}b_{k}{\text{log}}_{2}b_{k}$$

where M is the number of equally divided regions according to the distribution of the oscillation period and b_k _is the number of the distribution of periods of the kth region; N is the total number of the distribution of periods that are obtained through using the Latin sampling method [26] by a variation of the parameters 10% or 20%. (In our study, N = 1000). Obviously, 0 ≤ r ≤ 1, where r = 1 corresponds to perfect synchronization and perfect robustness (M = 1 and b_1 _= N), and r = 0 corresponds to no synchronization and poor robustness (M = N and b_k _= 1).

## Competing interests

The authors declare that they have no competing interests.

## Authors' contributions

XFZ designed the research and wrote the manuscript. WZ performed the methods and conducted the numerical experiments. All the authors read, edited and approved the final manuscript.

## Supplementary Material

Additional file 1 **The synchronization behavior of the coupled oscillators**. The coupled system achieved synchronization when the parameters were set as in Table 1. N is the number of cells. The character C refers to CDK1, P refers to PLK1, A refers to APC and R refers to the complex protein. Click here for file

Additional file 2 **The sensitivity of the coupled system to the perturbation of parameters**. (A) Sensitivity of CDK1 to the perturbation of parameters. (B) Sensitivity of PLK1 to the perturbation of parameters. (C) Sensitivity of APC to the perturbation of parameters. (D) Sensitivity of R to the perturbation of parameters. Click here for file

Additional file 3 **The bifurcation diagrams for K_1_, K_2_, α_1 _and α_3_**. (A) The bifurcation diagrams of the activation coefficients K_1 _in the Hill function. (B) The bifurcation diagrams of the activation coefficients K_2 _in the Hill function. (C) The bifurcation diagrams of the activation constants α_1_. (D) The bifurcation diagrams of the activation constants α_3_. Click here for file

Additional file 4 **The bifurcation diagrams for the degradation rates**. (A) The bifurcation diagrams of degradation rates β_2_. (B) The bifurcation diagrams of degradation rates β_3_. (C) The bifurcation diagrams of the degradation rate of complex protein R. (D) The coupled system achieved an asymptotically steady state when k_m _= 1.25. Click here for file

Additional file 5 **The bifurcation diagrams for the coupling parameters**. (A) The bifurcation diagrams for the activation coefficients K_L _in the Hill function. (B) The bifurcation diagrams for the activation coefficients K_a _in the Hill function. (C) The bifurcation diagrams for the coupling strength k. (D) The bifurcation diagram for the activation constant k_0_. Click here for file

Additional file 6 **The effects of K_1 _and K_3 _on the period and amplitude**. The above two diagrams show the effects of K_1 _on the period and amplitude of the coupled system when synchronization is achieved. The two diagrams below show the effects of K_3 _on the period and amplitude of the coupled system when synchronization is achieved. Click here for file

Additional file 7 **The effects of K_2 _on the period and amplitude**. The above two diagrams show the effects of K_2 _on the period and amplitude of the coupled system when synchronization is achieved at the first synchronization interval. The two diagrams below show the effects of K_2 _on the period and amplitude of the coupled system when synchronization is achieved at the second synchronization interval. Click here for file

Additional file 8 **The effects of α_1 _and α_3 _on the period and amplitude when synchronization is achieved**. The left two diagrams show the effects of α_1 _on the period and amplitude of the coupled system when synchronization is achieved at the first synchronization interval. The two diagrams on the right show the effects of α_3 _on the period and amplitude of the coupled system when synchronization is achieved at the second synchronization interval. Click here for file

Additional file 9 **The effects of parameters K_L_, K_a_, k and k_0 _on the period when synchronization is achieved**. With an increase in these parameters in their synchronization intervals, the oscillation periods for parameters K_L_, K_a _and k increase, but the oscillation period for parameter k_0 _decreases. Click here for file

Additional file 10 **The effects of parameters K_L_, K_a_, k and k_0 _on the amplitude when achieved synchronization**. With an increase in these parameters in their synchronization intervals, the oscillation amplitudes for parameters K_L _and K_a _increase, but the oscillation amplitudes for parameters k and k_0 _decreases. Click here for file
