# Supplementary figures and images for: Synchronization ability of coupled cell-cycle oscillators in changing environments
Source: BMC Syst Biol. 2012 Jul 16;6(Suppl 1):S13. doi: 10.1186/1752-0509-6-S1-S13 (PMC3403058; doi:10.1186/1752-0509-6-S1-S13)

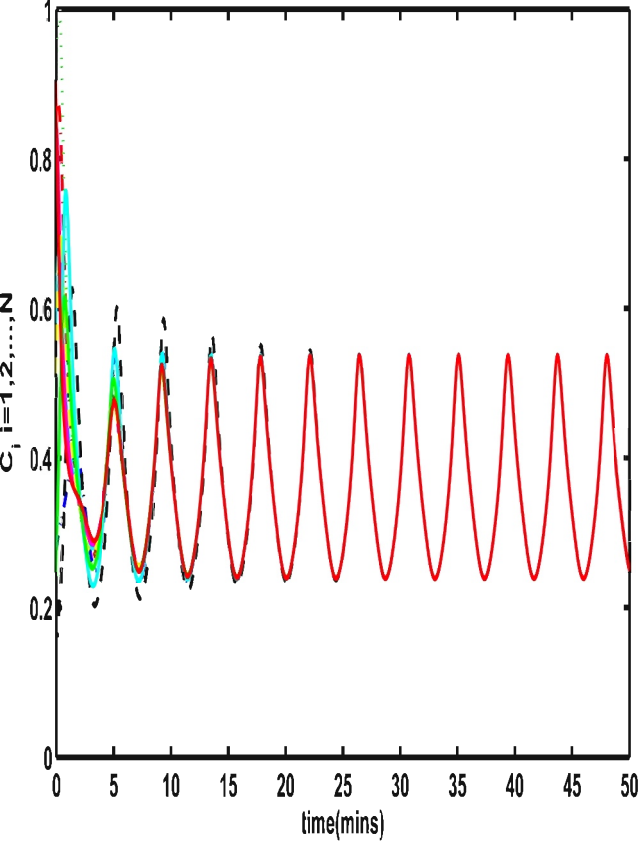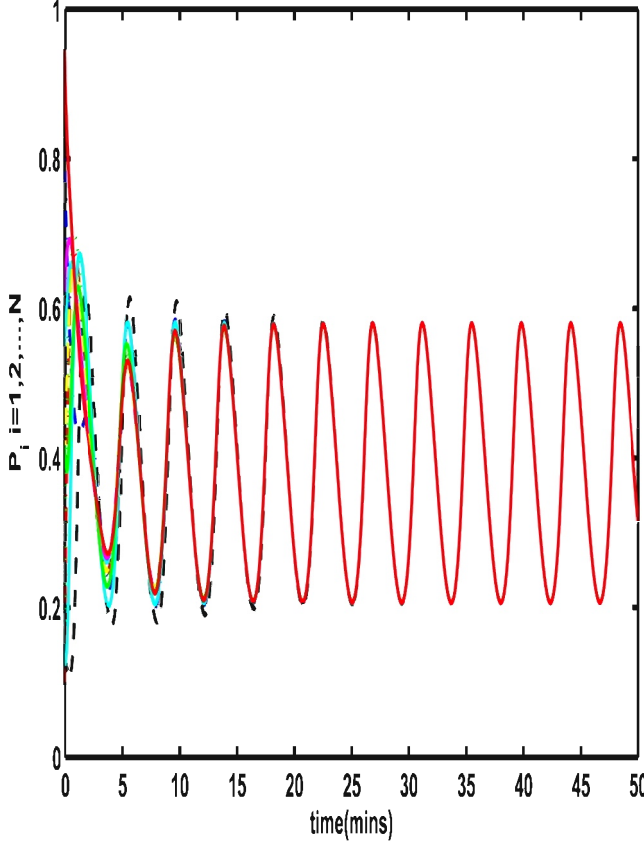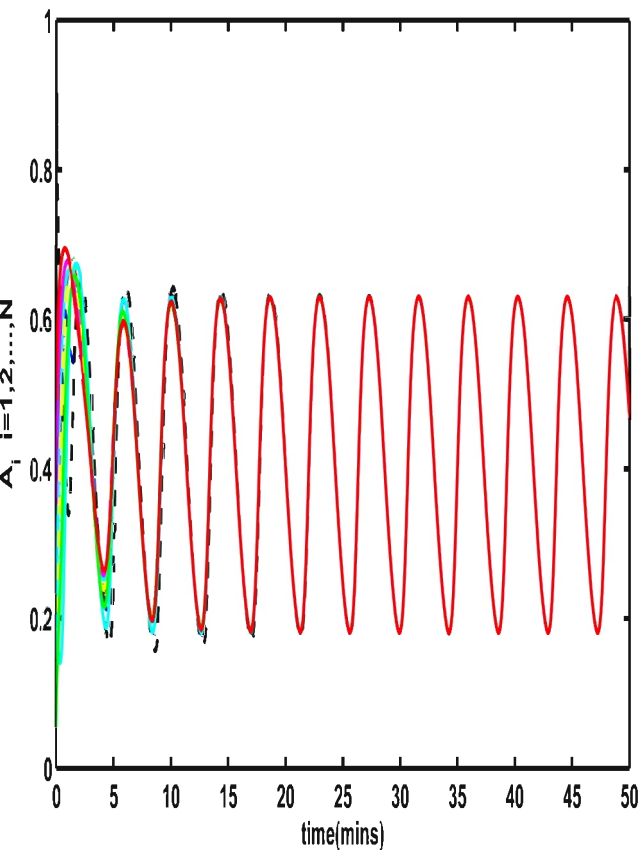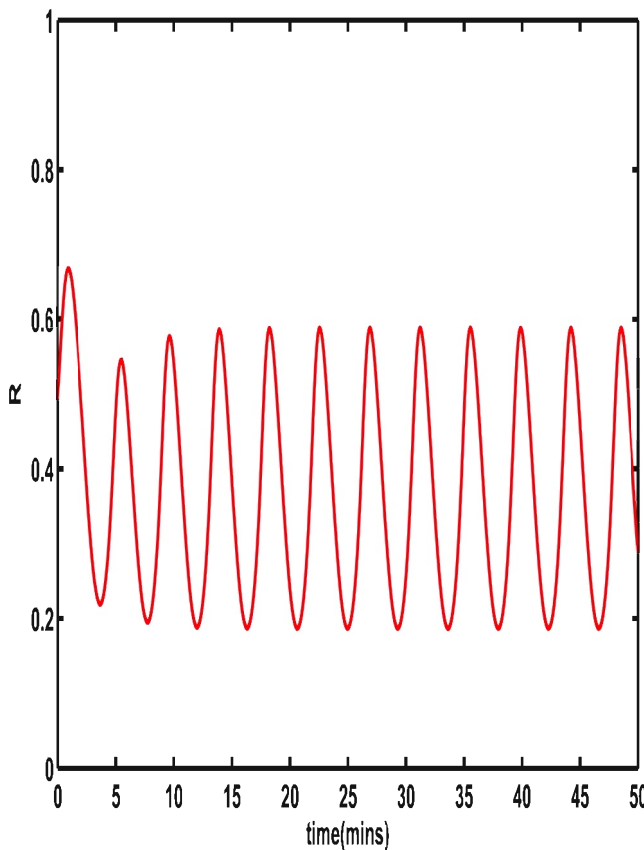

Supplement: Additional file 1 — The synchronization behavior of the coupled oscillators. The coupled system achieved synchronization when the parameters were set as in Table 1. N is the number of cells. The character C refers to CDK1, P refers to PLK1, A refers to APC and R refers to the complex protein. [file 1752-0509-6-S1-S13-S1.pdf]

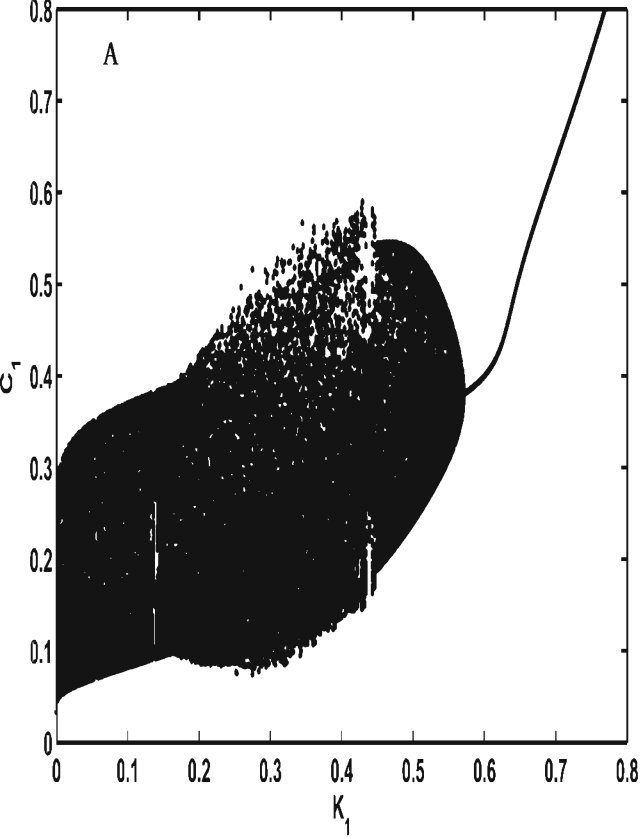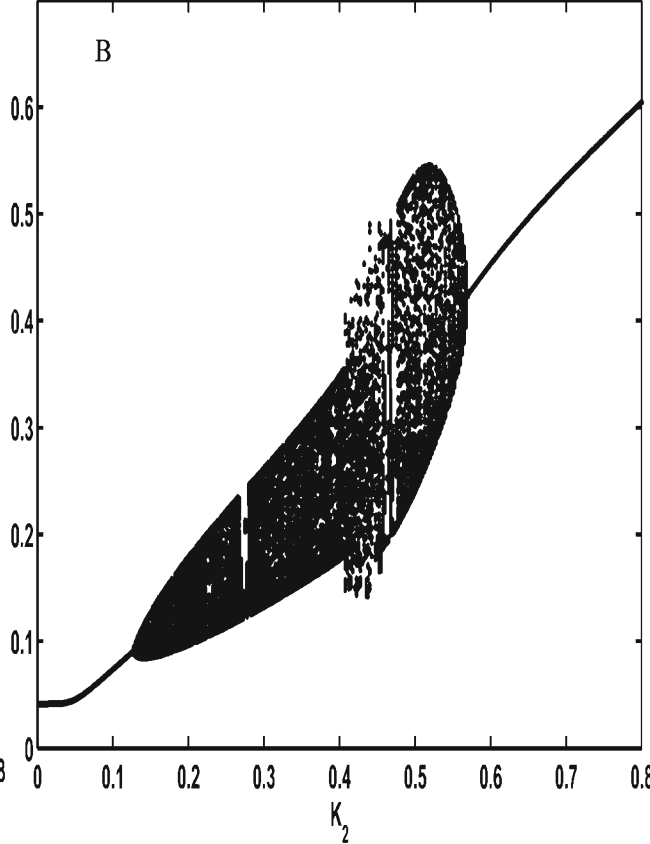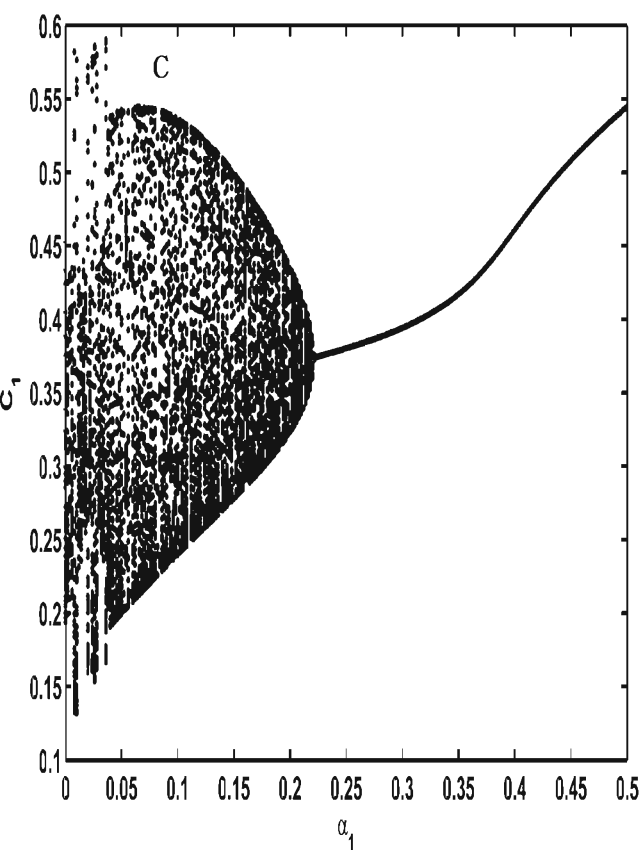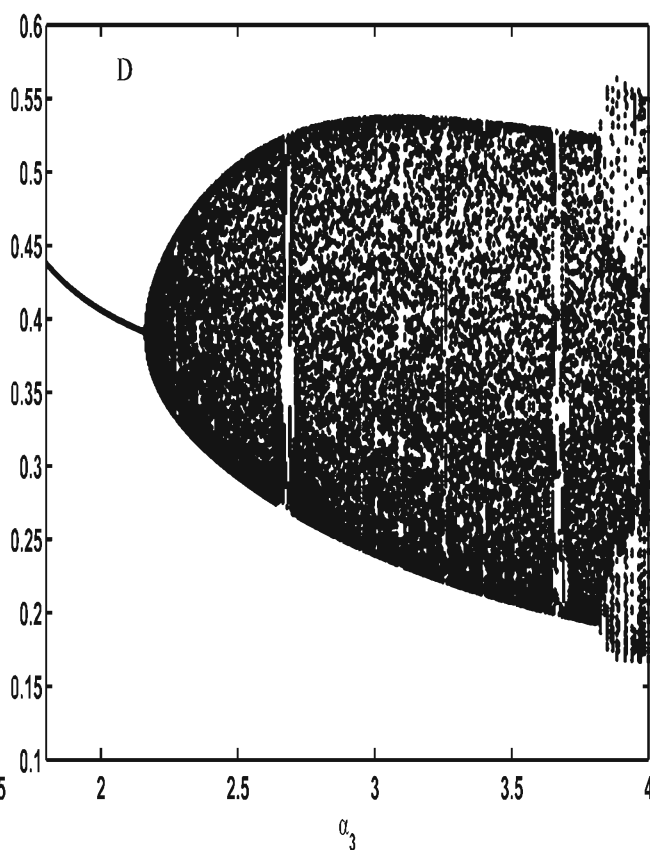

Supplement: Additional file 3 — The bifurcation diagrams for K1, K2, α1 and α3. (A) The bifurcation diagrams of the activation coefficients K1 in the Hill function. (B) The bifurcation diagrams of the activation coefficients K2 in the Hill function. (C) The bifurcation diagrams of the activation constants α1. (D) The bifurcation diagrams of the activation constants α3. [file 1752-0509-6-S1-S13-S3.pdf]

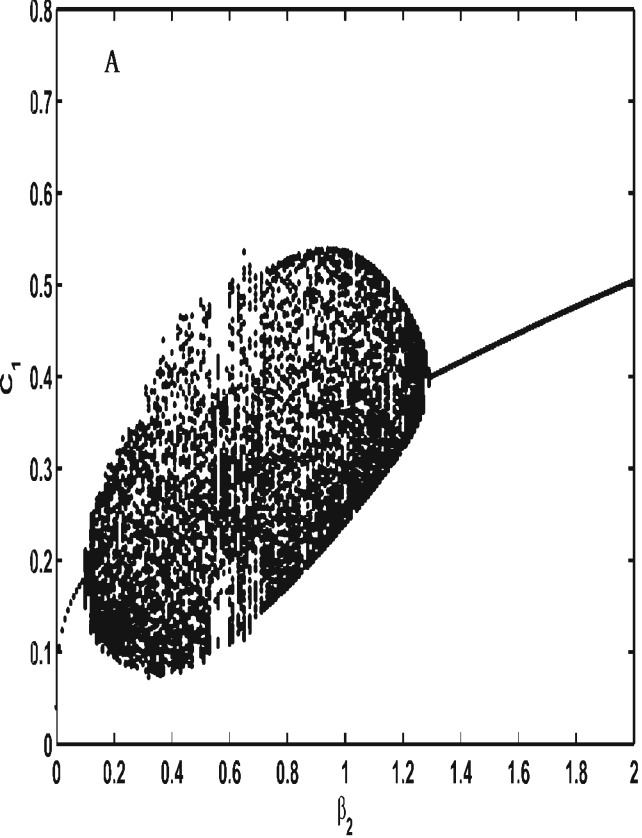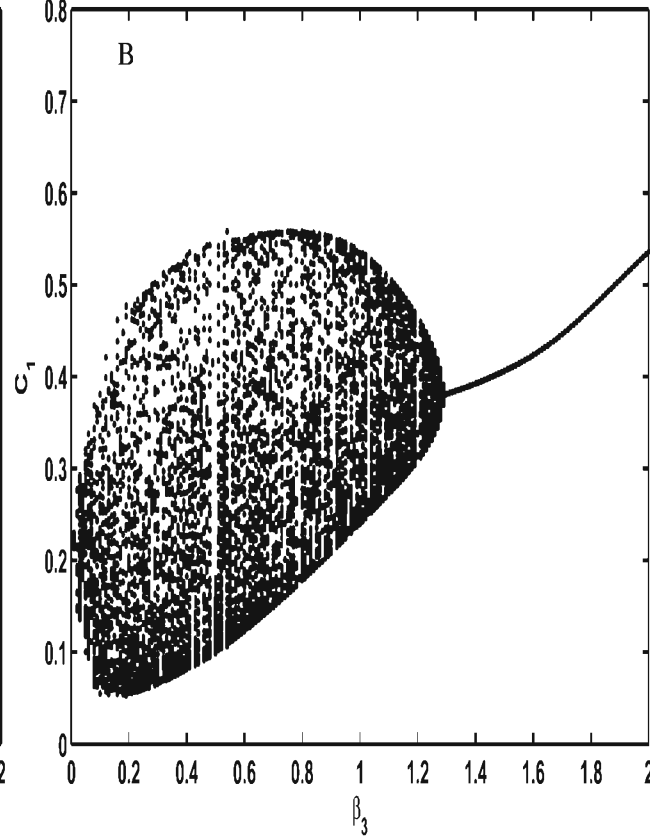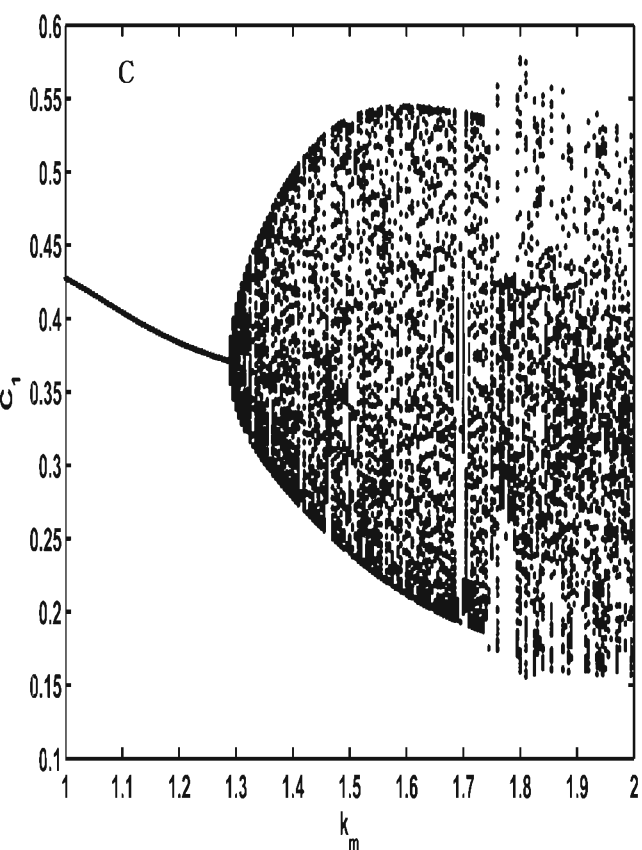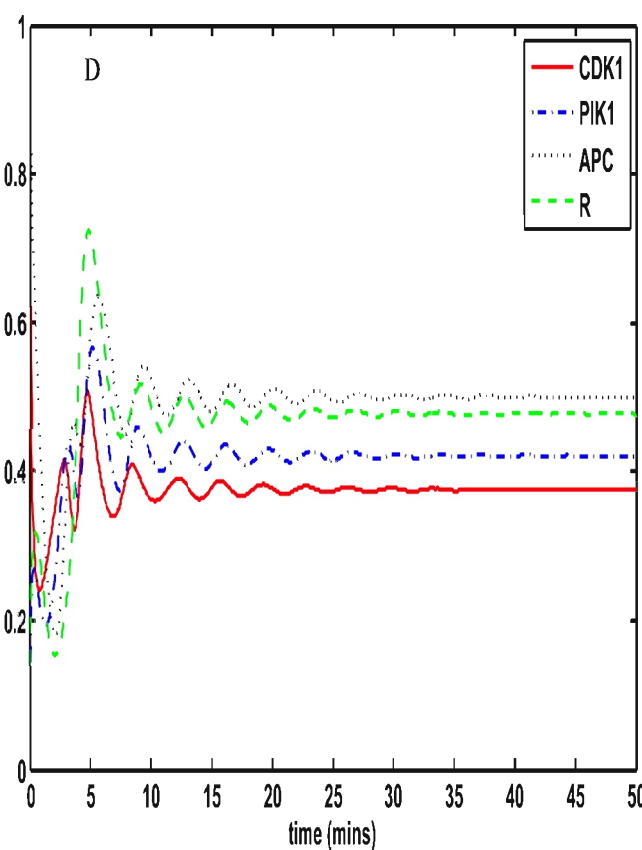

Supplement: Additional file 4 — The bifurcation diagrams for the degradation rates. (A) The bifurcation diagrams of degradation rates β2. (B) The bifurcation diagrams of degradation rates β3. (C) The bifurcation diagrams of the degradation rate of complex protein R. (D) The coupled system achieved an asymptotically steady state when km = 1.25. [file 1752-0509-6-S1-S13-S4.pdf]

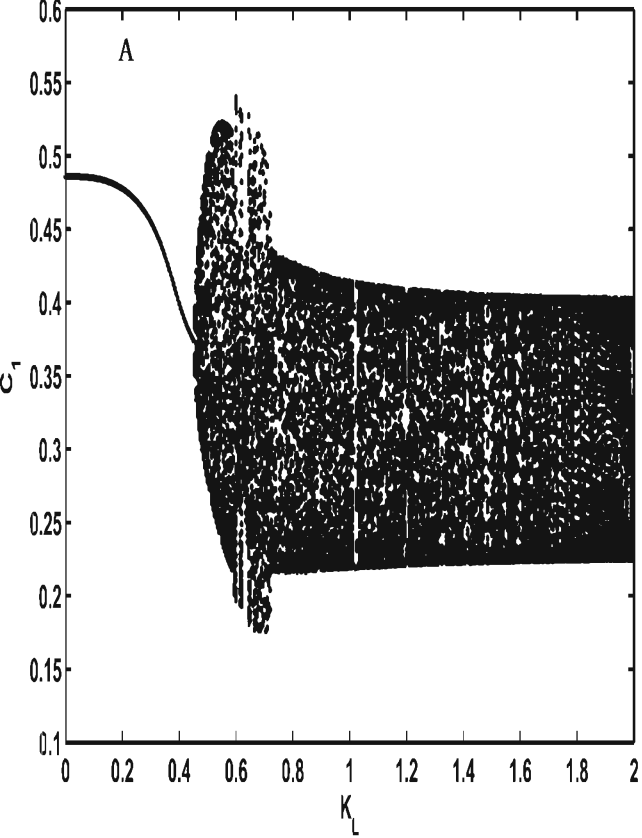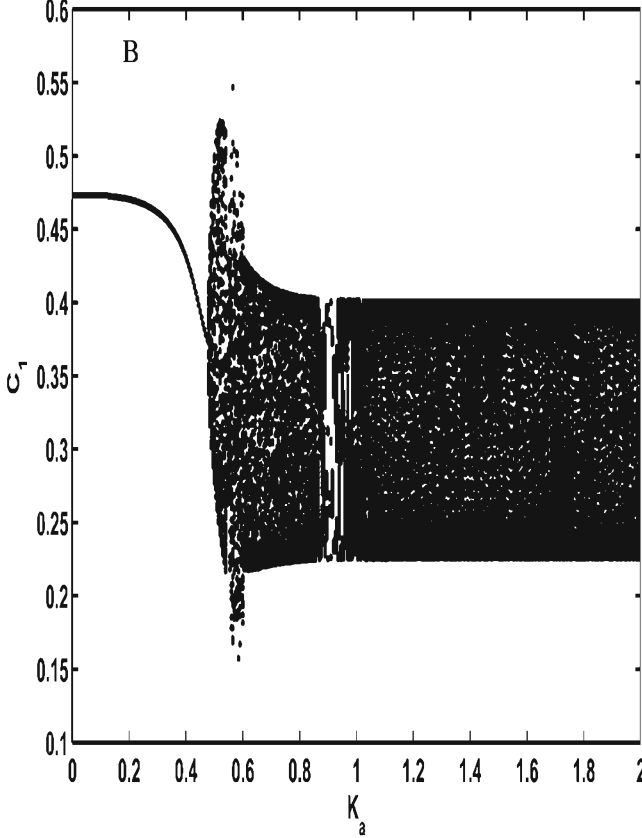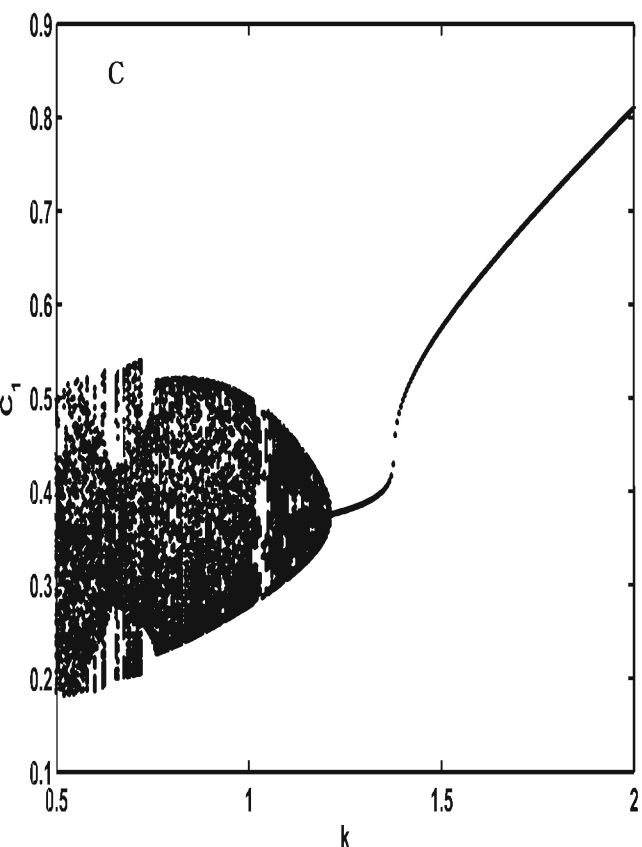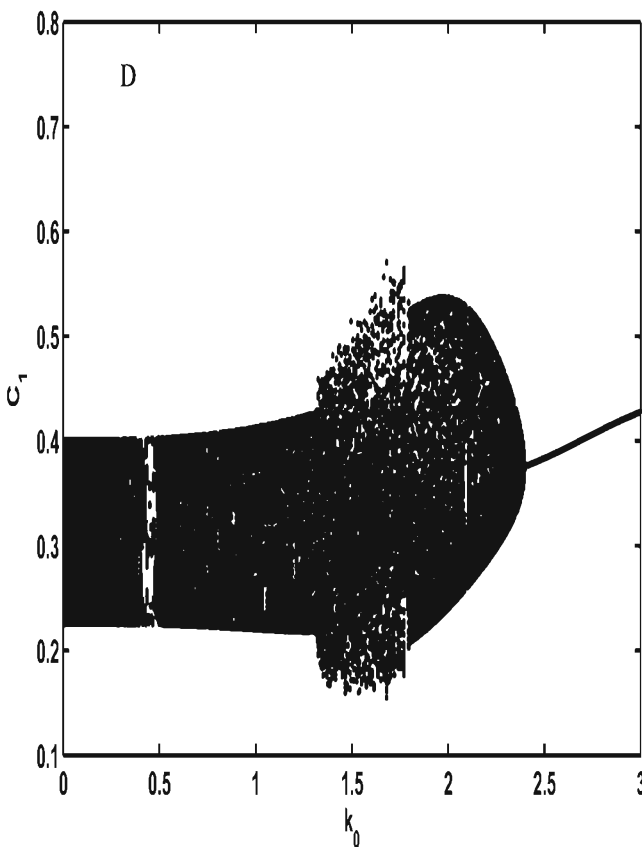

Supplement: Additional file 5 — The bifurcation diagrams for the coupling parameters. (A) The bifurcation diagrams for the activation coefficients KL in the Hill function. (B) The bifurcation diagrams for the activation coefficients Ka in the Hill function. (C) The bifurcation diagrams for the coupling strength k. (D) The bifurcation diagram for the activation constant k0. [file 1752-0509-6-S1-S13-S5.pdf]

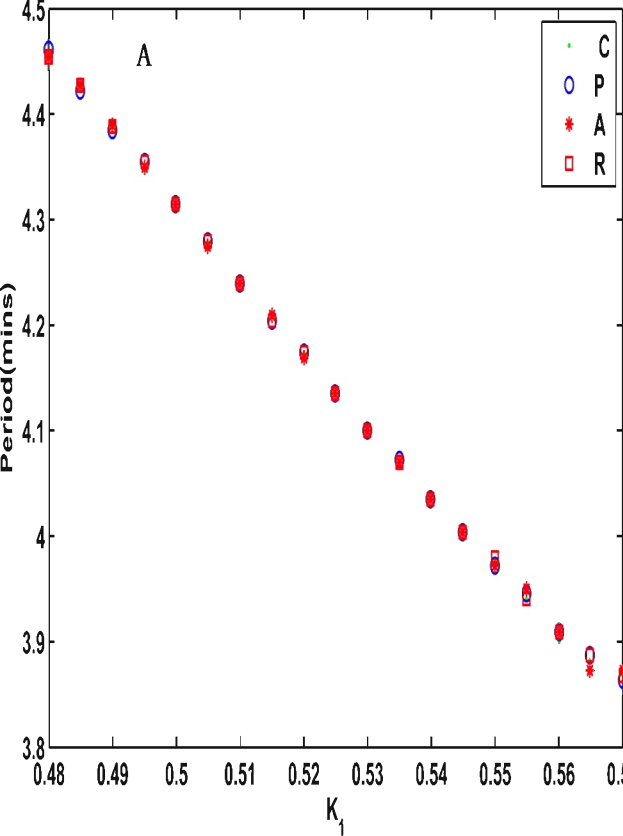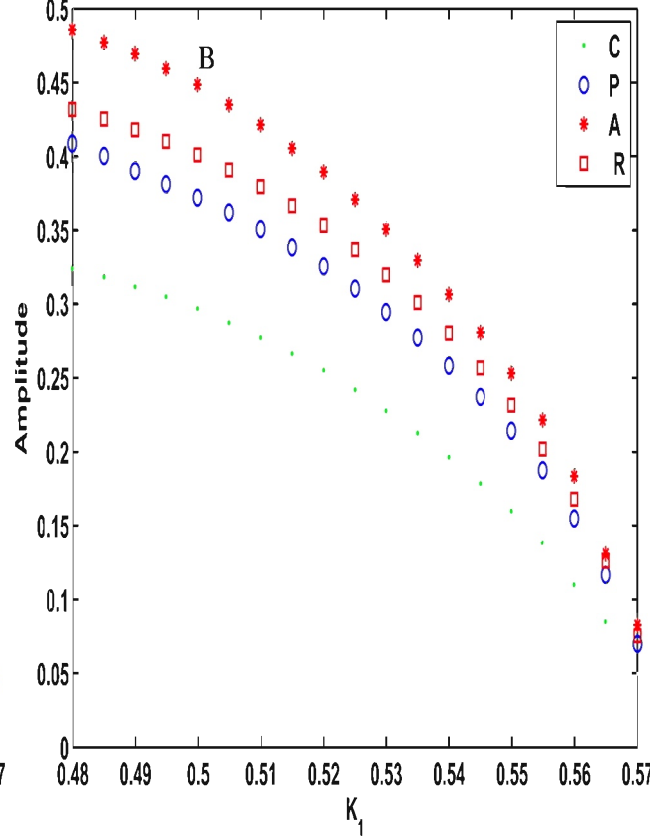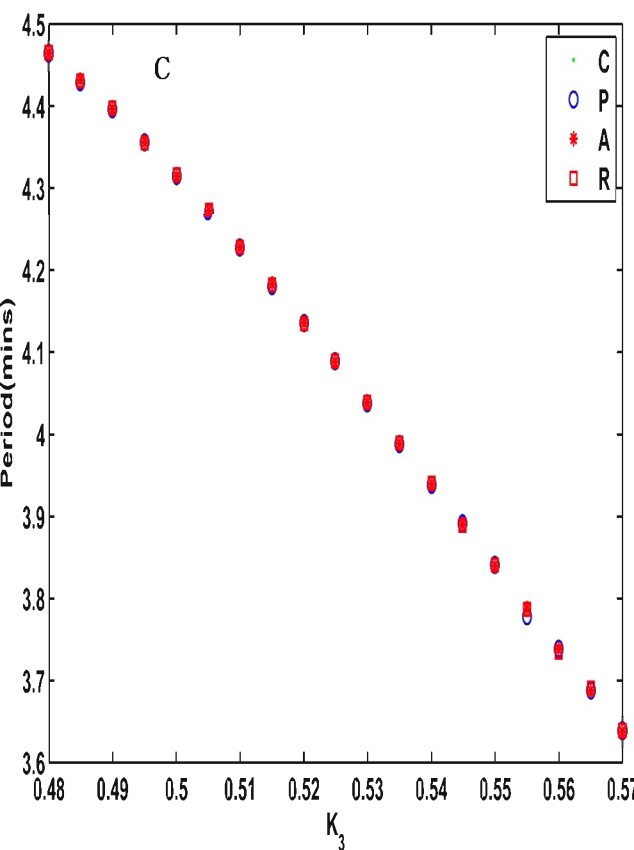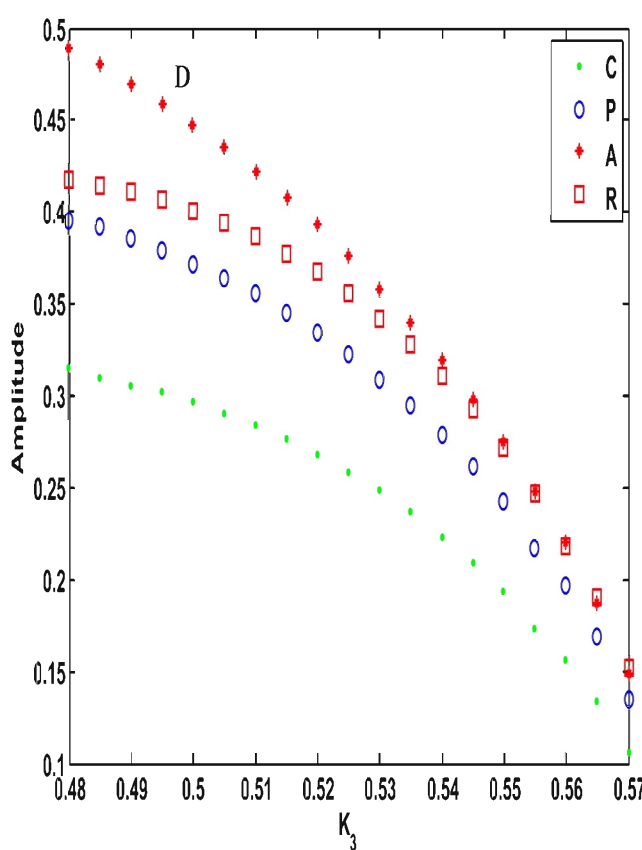

Supplement: Additional file 6 — The effects of K1 and K3 on the period and amplitude. The above two diagrams show the effects of K1 on the period and amplitude of the coupled system when synchronization is achieved. The two diagrams below show the effects of K3 on the period and amplitude of the coupled system when synchronization is achieved. [file 1752-0509-6-S1-S13-S6.pdf]

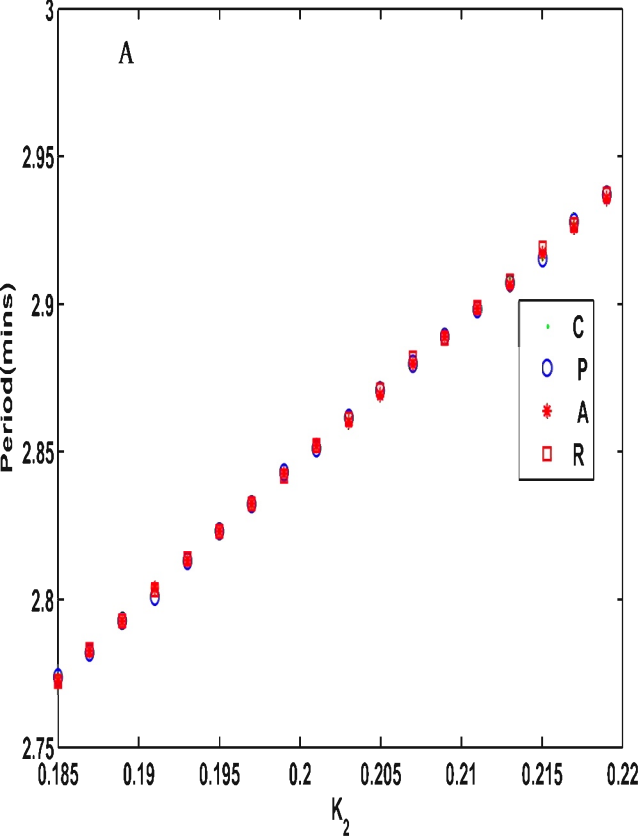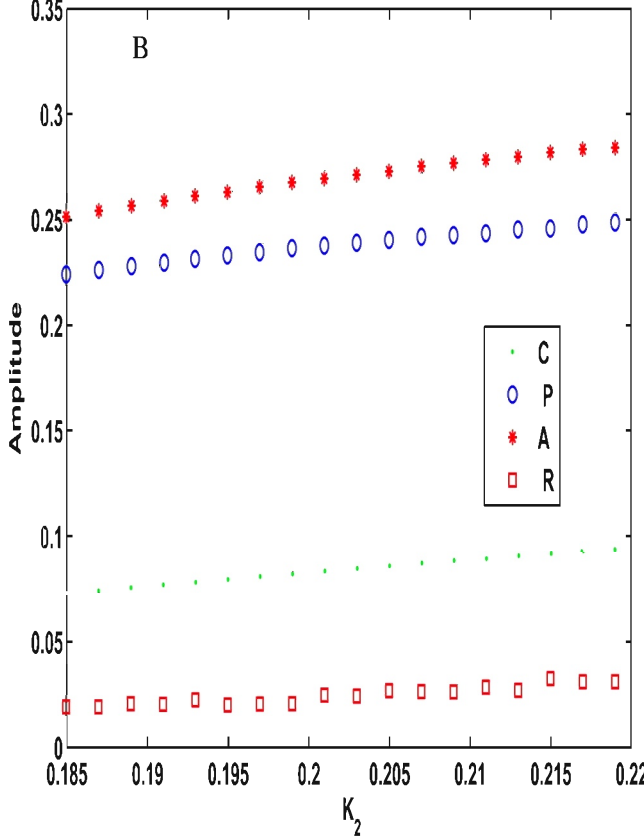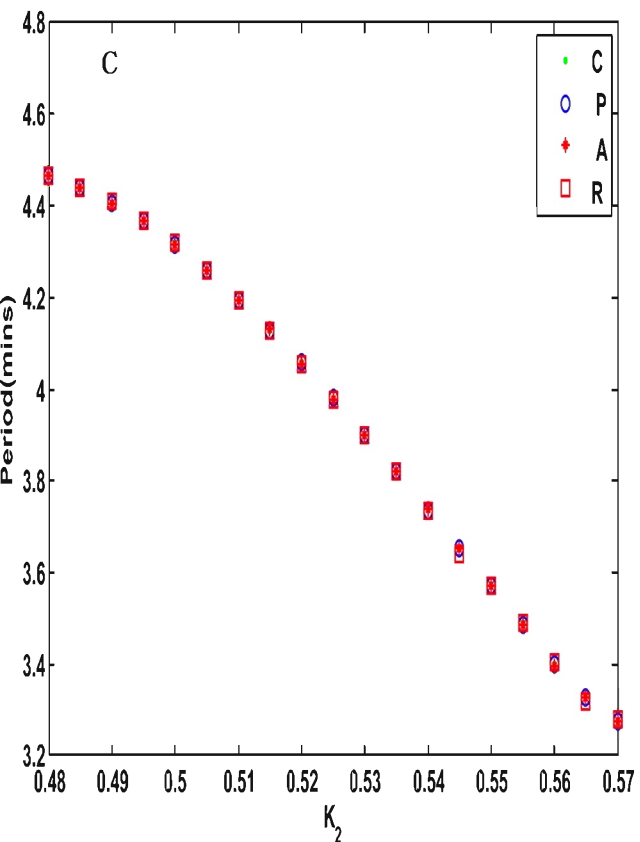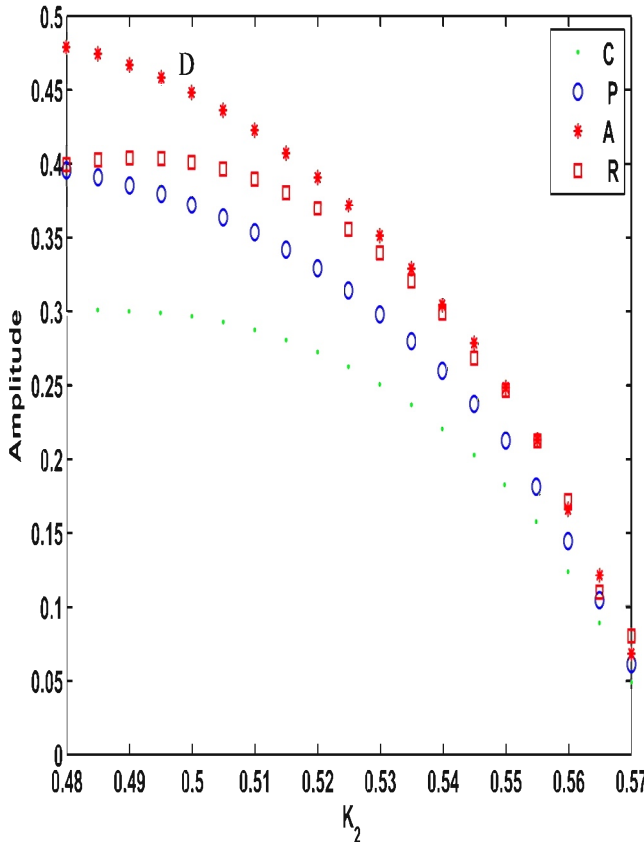

Supplement: Additional file 7 — The effects of K2 on the period and amplitude. The above two diagrams show the effects of K2 on the period and amplitude of the coupled system when synchronization is achieved at the first synchronization interval. The two diagrams below show the effects of K2 on the period and amplitude of the coupled system when synchronization is achieved at the second synchronization interval. [file 1752-0509-6-S1-S13-S7.pdf]

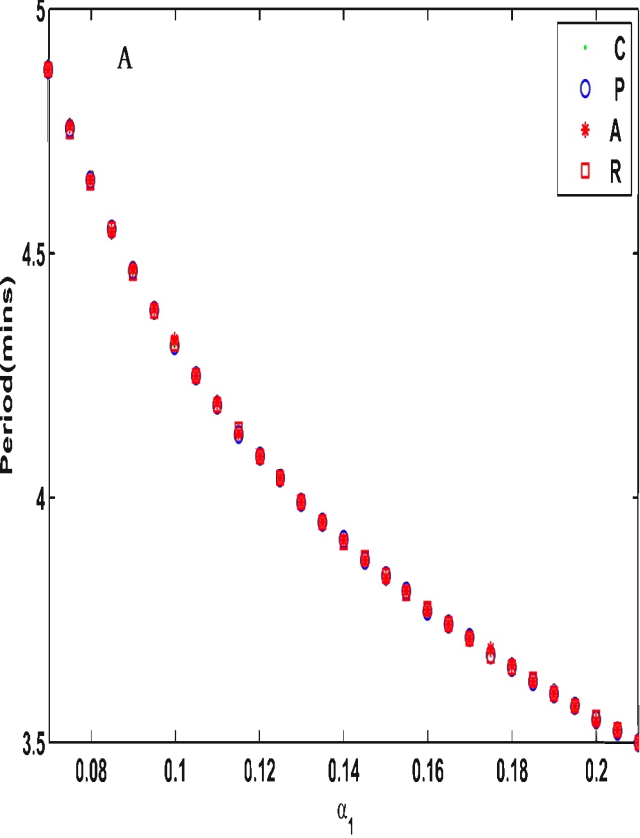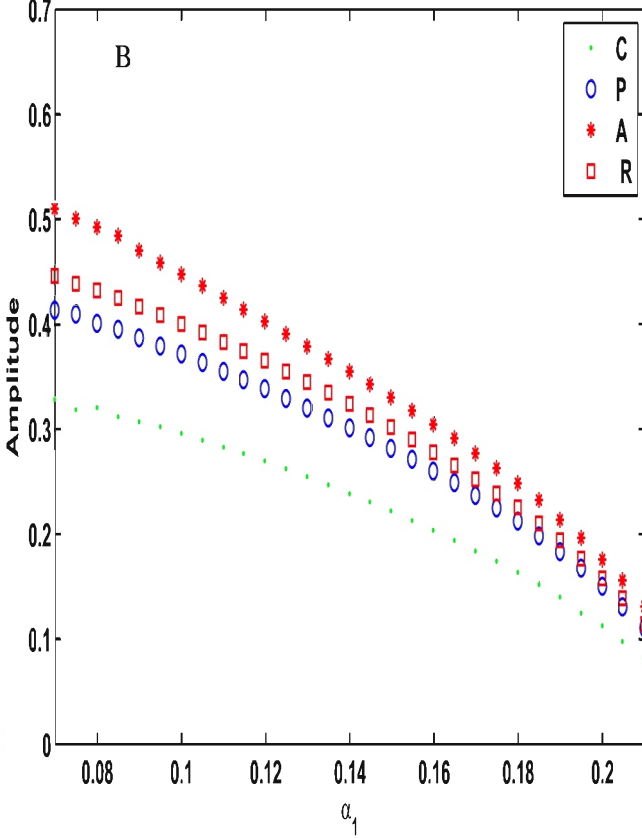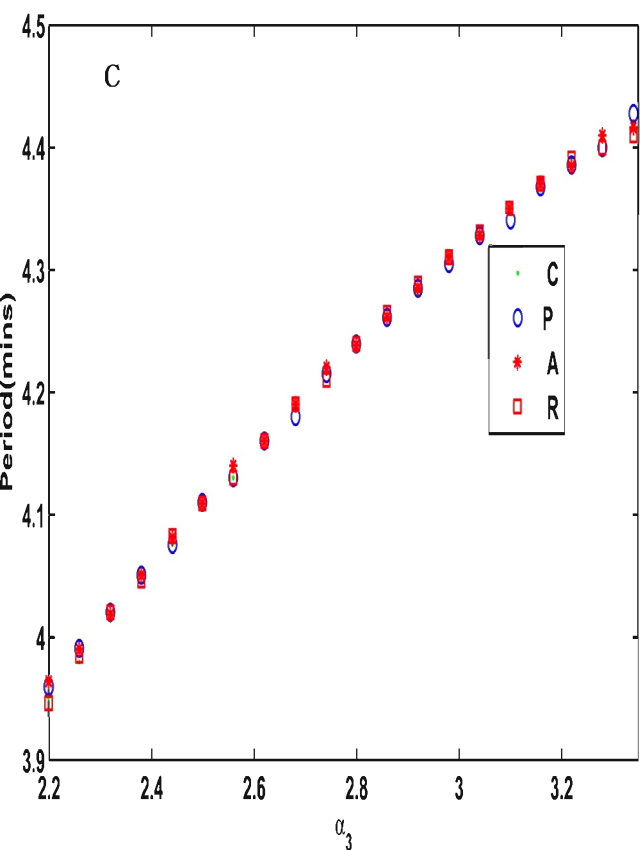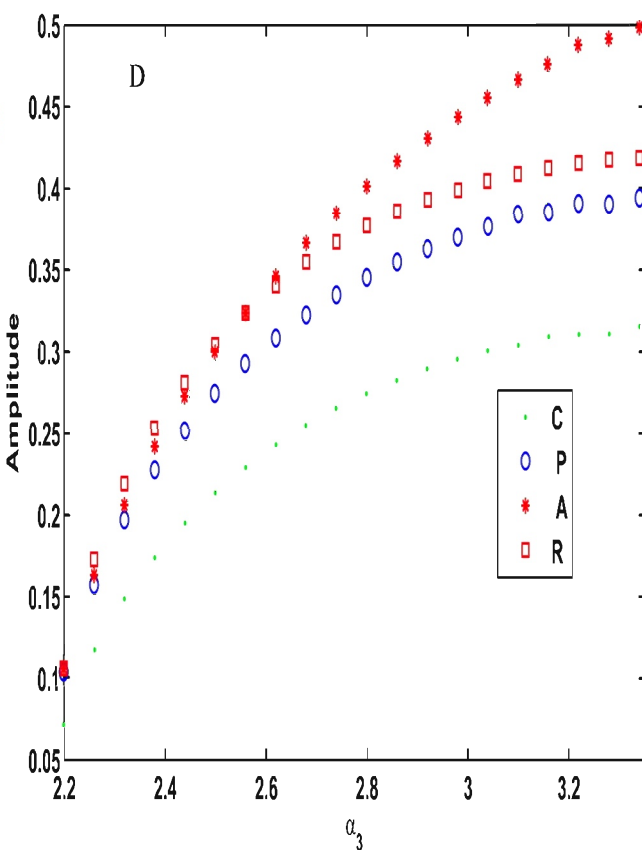

Supplement: Additional file 8 — The effects of α1 and α3 on the period and amplitude when synchronization is achieved. The left two diagrams show the effects of α1 on the period and amplitude of the coupled system when synchronization is achieved at the first synchronization interval. The two diagrams on the right show the effects of α3 on the period and amplitude of the coupled system when synchronization is achieved at the second synchronization interval. [file 1752-0509-6-S1-S13-S8.pdf]

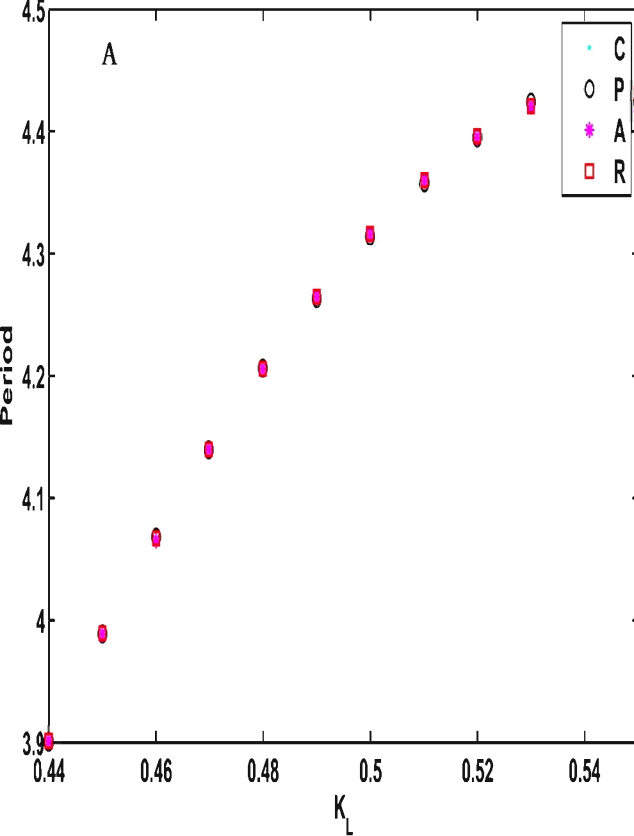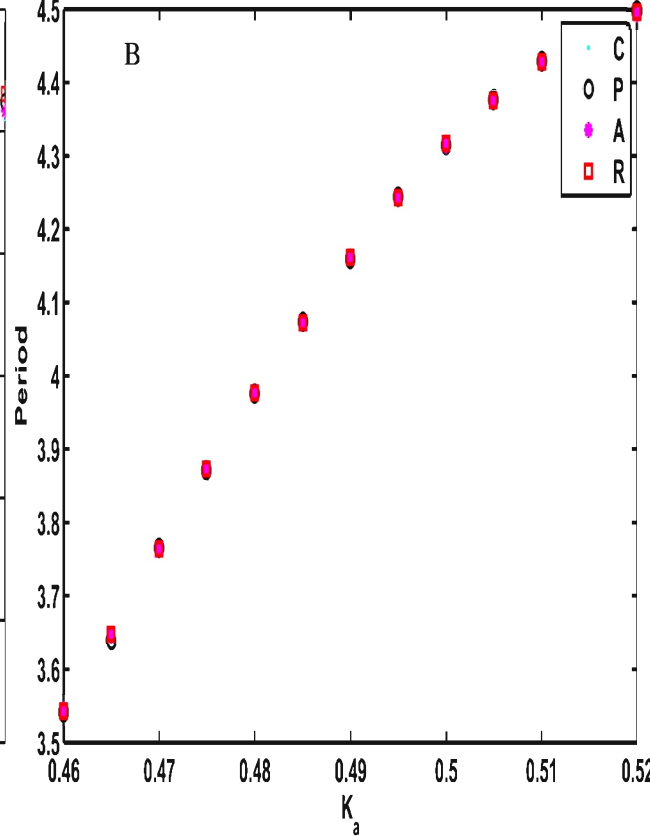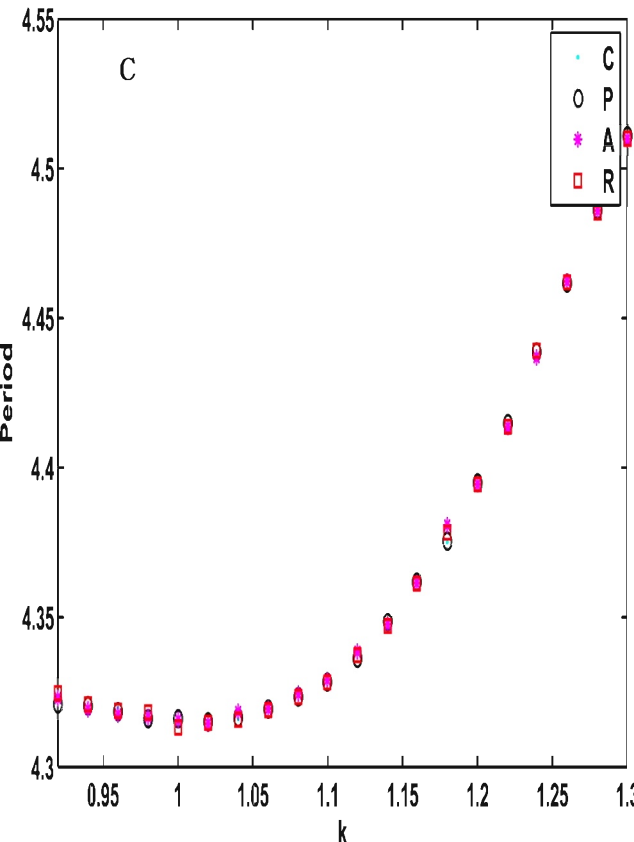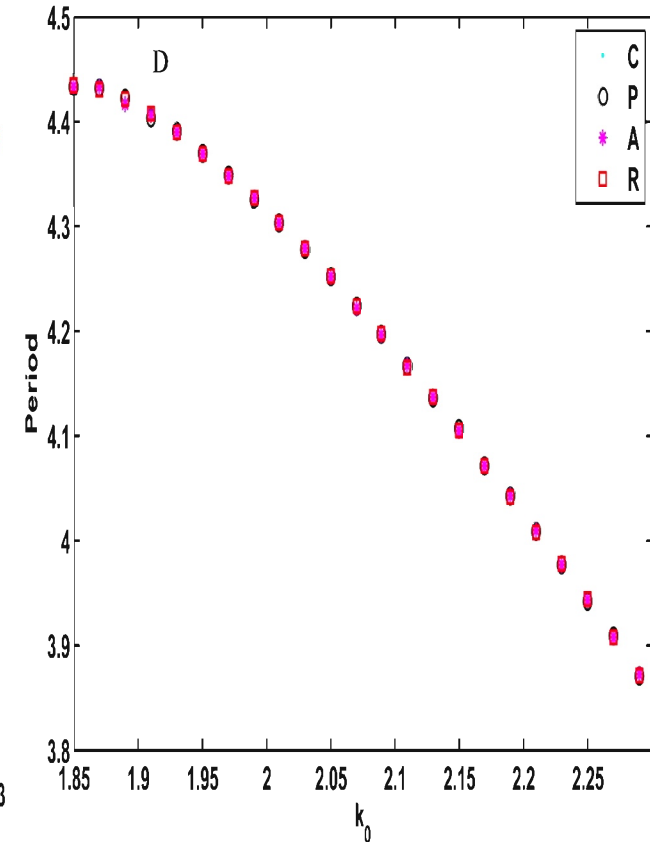

Supplement: Additional file 9 — The effects of parameters KL, Ka, k and k0 on the period when synchronization is achieved. With an increase in these parameters in their synchronization intervals, the oscillation periods for parameters KL, Ka and k increase, but the oscillation period for parameter k0 decreases. [file 1752-0509-6-S1-S13-S9.pdf]

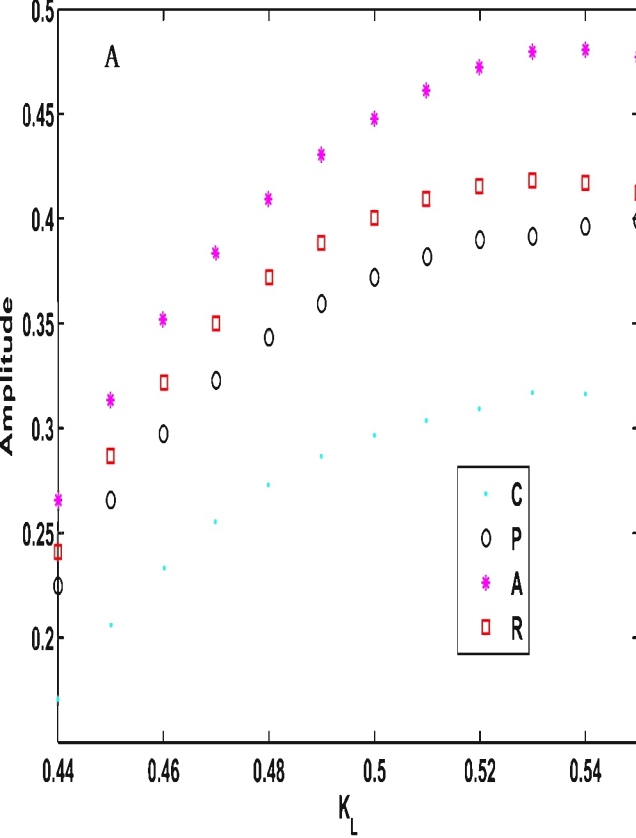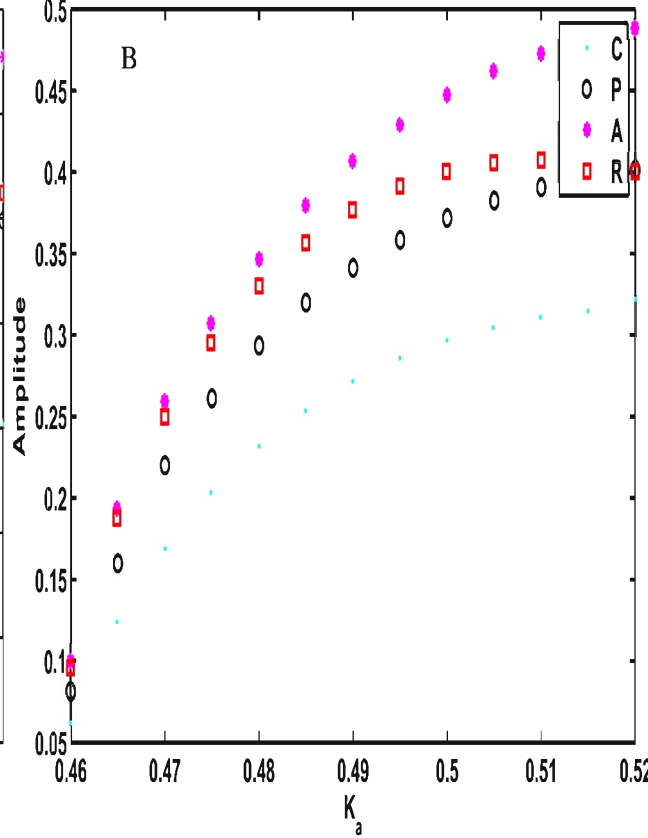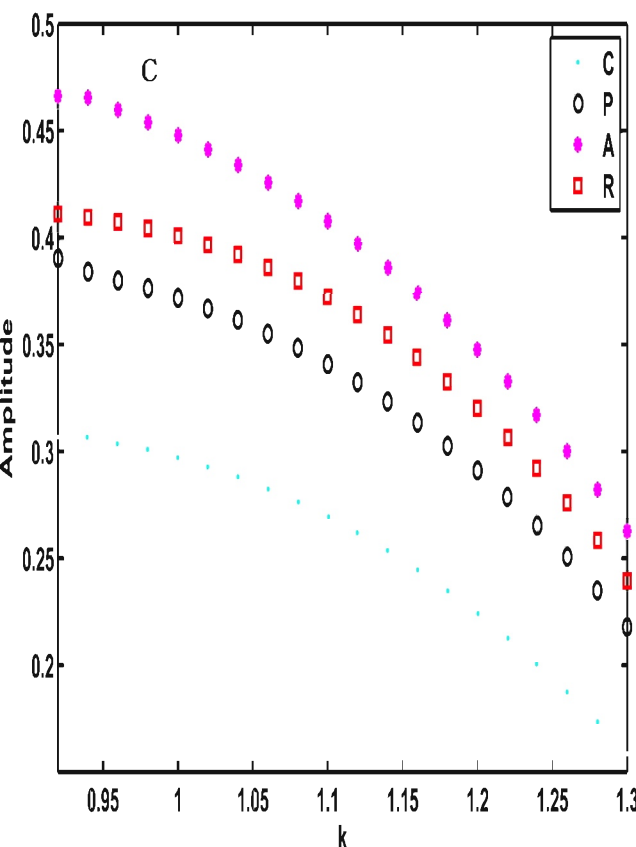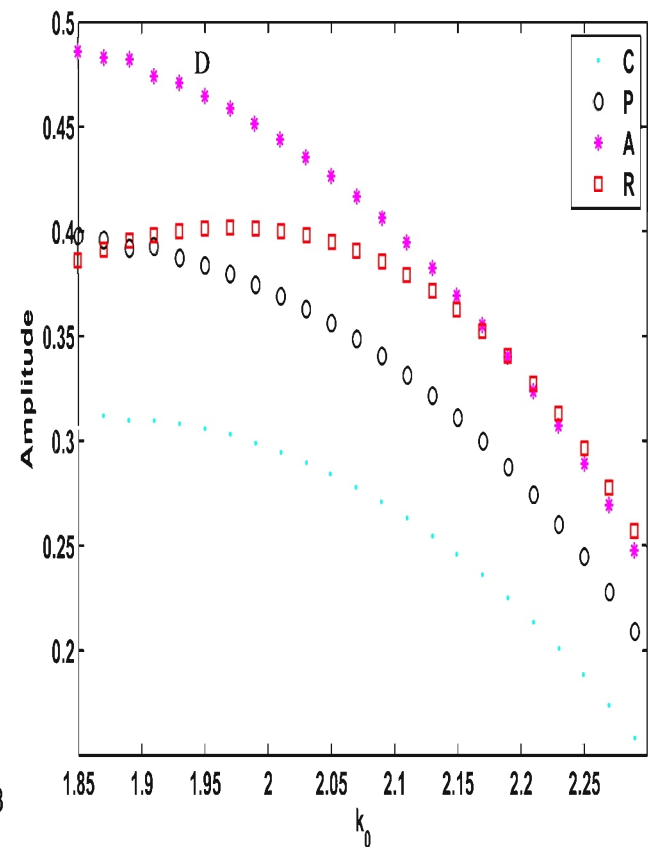

Supplement: Additional file 10 — The effects of parameters KL, Ka, k and k0 on the amplitude when achieved synchronization. With an increase in these parameters in their synchronization intervals, the oscillation amplitudes for parameters KL and Ka increase, but the oscillation amplitudes for parameters k and k0 decreases. [file 1752-0509-6-S1-S13-S10.pdf]
