# Supplementary material for: Synchronization ability of coupled cell-cycle oscillators in changing environments
Source: BMC Syst Biol. 2012 Jul 16;6(Suppl 1):S13. doi: 10.1186/1752-0509-6-S1-S13 (PMC3403058; doi:10.1186/1752-0509-6-S1-S13)
Supplement: Additional file 2 — The sensitivity of the coupled system to the perturbation of parameters. (A) Sensitivity of CDK1 to the perturbation of parameters. (B) Sensitivity of PLK1 to the perturbation of parameters. (C) Sensitivity of APC to the perturbation of parameters. (D) Sensitivity of R to the perturbation of parameters. [file 1752-0509-6-S1-S13-S2.pdf]

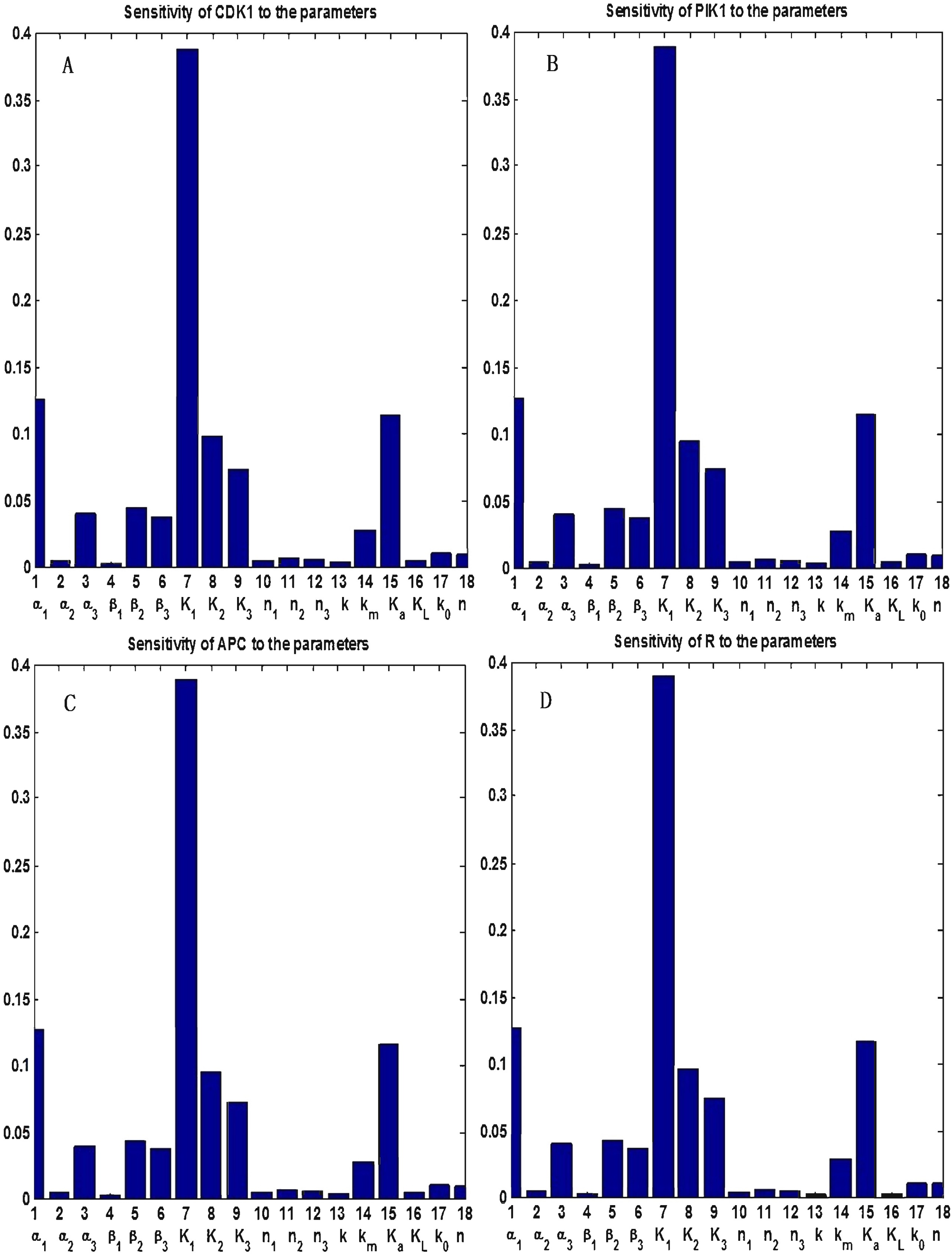
Sensitivity of CDK1 to the parameters
A
Sensitivity of PIK1 to the parameters
B
Sensitivity of APC to the parameters
C
Sensitivity of R to the parameters
D
0.4
0.35
0.3
0.25
0.2
0.15
0.1
0.05
0
1 2 3 4 5 6 7 8 9 10 11 12 13 14 15 16 17 18
α1 α2 α3 β1 β2 β3 K1 K2 K3 n1 n2 n3 k km Ka KL k0 n
